# Pathogenicity and virulence of *Clostridium botulinum*

**DOI:** 10.1080/21505594.2023.2205251

**Published:** 2023-05-08

**Authors:** Alexander M. Rawson, Andrew W. Dempster, Christopher M. Humphreys, Nigel P. Minton

**Affiliations:** Clostridia Research Group, BBSRC/EPSRC Synthetic Biology Research Centre (SBRC), School of Life Sciences, The Biodiscovery Institute, The University of Nottingham, Nottingham, UK

**Keywords:** *Clostridium botulinum*, botulism, neurotoxin (BoNT), sporulation, germination, food contamination, therapeutics

## Abstract

*Clostridium botulinum*, a polyphyletic Gram-positive taxon of bacteria, is classified purely by their ability to produce botulinum neurotoxin (BoNT). BoNT is the primary virulence factor and the causative agent of botulism. A potentially fatal disease, botulism is classically characterized by a symmetrical descending flaccid paralysis, which is left untreated can lead to respiratory failure and death. Botulism cases are classified into three main forms dependent on the nature of intoxication; foodborne, wound and infant. The BoNT, regarded as the most potent biological substance known, is a zinc metalloprotease that specifically cleaves SNARE proteins at neuromuscular junctions, preventing exocytosis of neurotransmitters, leading to muscle paralysis. The BoNT is now used to treat numerous medical conditions caused by overactive or spastic muscles and is extensively used in the cosmetic industry due to its high specificity and the exceedingly small doses needed to exert long-lasting pharmacological effects. Additionally, the ability to form endospores is critical to the pathogenicity of the bacteria. Disease transmission is often facilitated via the metabolically dormant spores that are highly resistant to environment stresses, allowing persistence in the environment in unfavourable conditions. Infant and wound botulism infections are initiated upon germination of the spores into neurotoxin producing vegetative cells, whereas foodborne botulism is attributed to ingestion of preformed BoNT. *C.* *botulinum* is a saprophytic bacterium, thought to have evolved its potent neurotoxin to establish a source of nutrients by killing its host.

## Graphical abstract

**Figure:**
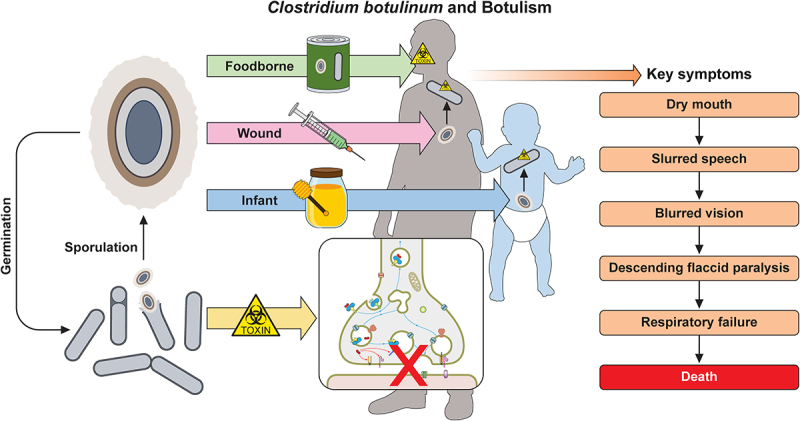

## Introduction – the organism

*Clostridium botulinum* is a Gram-positive, anaerobic, endospore-forming bacillus responsible for botulism, a severe neuroparalytic disease that affects humans and vertebrate animals [1–3]. The causative agent is botulinum neurotoxin (BoNT), renowned as the most potent biological substance known to humankind [4,5]. A metalloprotease, BoNT specifically cleaves the Soluble N-Ethylmaleimide-Sensitive Factor Attachment Protein Receptor (SNARE) proteins in postsynaptic nerve terminals, preventing the release of neurotransmitter and blocking neural transmission to effector muscles [6].

Ubiquitous in nature, humans and animals routinely encounter this bacterium in the form of endospores [7]. Formed under stressful conditions to allow survival, spores are a characteristic trait of the genus *Clostridium*, members of which includes other notable pathogens, such as *Clostridium difficile* (now reclassified as *Clostridioides difficile), Clostridium tetani* and *Clostridium perfringens*. These pathogens are the causative agents of chronic diarrhoea, tetanus, and gas gangrene, respectively, [8,9]. The spores of *C. botulinum* usually present no threat to people unless they germinate and form neurotoxin-producing, vegetative cells. As the healthy human digestive system is not conducive to spore germination, these routinely pass through our bodies and are excreted without causing any harm, except for infant and adult gut dysbiosis botulism cases [10].

*Clostridium botulinum* has become one of the most notorious organisms in medical history, not only for outbreaks of fatal botulism cases but also for the development of pharmaceutical applications of the botulinum neurotoxin [11]. Commonly used for treatment of numerous medical conditions in fields of dermatology, ophthalmology and neurology, the botulinum neurotoxins are now perhaps most famous for cosmetic applications [12–15]. Consequently, the toxicology and pharmacology of the BoNTs is well studied, but less is known of the physiology of the bacteria responsible for their production.

### C. *botulinum* - A historical perspective

Since humans have stored foods, *C. botulinum* and its neurotoxin have been problematic for causing cases of foodborne botulism [16]. Following the poverty caused by the Napoleonic war in Europe in the late 1700s a mysterious illness attributed to poor food production led to many deaths [17]. It wasn’t until in the early 18^th^ century that the source was postulated. Across Southwest Germany, an increasing number of fatal food poisoning cases were reported after consumption of a traditional uncooked blood sausage meal. This led a young local medical officer and poet, Justinus Kerner, now known as the godfather of botulinum research, to connect the blood sausage to the paralytic illness. He published the first complete descriptions of botulism in 1820 [18]. He termed the ailment “sausage poisoning” making it a reportable disease, and was the first to suggest the cause as a biological poison, performing experiments with the poison upon himself. Kerner even postulated the use of the “poison” to treat a variety of diseases in 1822, which was realised over 150 years after it was first envisaged [18,19].

In December 1895, a group of musicians, having just played at a funeral in Belgium, shared a customary meal of smoked and pickled ham. They all subsequently developed paralytic symptoms and three out of the 34 band members died [20]. Physician, Emile van Ermengem, investigated the outbreak and isolated the same anaerobic bacteria from both samples of infected ham and the victim’s corpses. Ermengem named the bacteria *Bacillus botulinus* (botulinus translating to sausage in Latin) and demonstrated its production of an unknown toxin [21]. In 1917, the anaerobic bacilli were reclassified into the genus *Clostridium* (from the Greek word “Kloster,” meaning spindle shape) and was subsequently renamed to *Clostridium botulinum* [22].

Georgina Burke was responsible for the letter designation of different serotypes in 1919 (BoNT/A and B) [23]. It took until the 1920s for the toxin to be purified when Herman Sommer managed to form an acid precipitate of BoNT/A, and in 1946, the microbiologist Carl Lamanna produced a crystallized form [24,25]. Following the crystallization Burgen’s group was the first to discover that BoNT blocked the release of the neurotransmitter acetylcholine at the neuromuscular junction [26]. Some 43 years later, Schiavo identified BoNT and the tetanus neurotoxin toxin (TeNT) as metalloproteases, which cleave SNARE proteins within presynaptic nerve terminals [27].

Although the potential of the toxin for medical applications was acknowledged by Kerner over 200 years ago, the use of BoNT as a therapeutic only began to be realised in the 1980s [28,29]. Ophthalmologist Alan Scott was the first to successfully demonstrate its therapeutic use as a treatment for strabismus (eye misalignment) through a collaboration with Edward Schantz at the University of Wisconsin, who supplied the purified the BoNT/A needed for the early clinical studies [30–32]. In 1989, the FDA (Food and Drug Administration) gave the approval of the BoNT/A orphan drug “Oculinum” for the treatment of strabismus, blepharospasm and hemifacial spasms [28]. The pharmaceutical company Allergan (Irvine, CA) acquired the rights to Oculinum soon after and later changed its name to the well-known Botox® (onabotulinumtoxinA) [33,34].

In parallel to Scott’s developments in the US, a partnership between biotechnology company Porton International (Ipsen Biopharm Ltd purchased the successor company) and the Centre for Applied Microbiology and Research (CAMR) at Porton Down were developing Dysport (abobotulinumtoxinA) in the United Kingdom. Shortly after the Oculinum approval, Dysport® (Dystonia/Porton Down) was approved in Europe in 1990 for the treatment of dystonia and was later the FDA approved in 2009 [34]. Four other BoNT products are currently FDA approved; incobotulinumtoxinA (Xeomin®; Merz Pharmaceuticals, Frankfurt, Germany), prabotulinumtoxinA (Jeuveau®; Evolus Inc, CA, USA), daxibotulinumtoxinA (DAXXIFY™; Revance Therapeutics Inc, TN, USA) and BoNT/B preparation rimabotulinumtoxinB (Myobloc® in the USA; Supernus Pharmaceuticals Inc, MD, USA/Neurobloc® in Europe; Sloan Pharma, Switzerland) [35]. Other BoNT/A formulations are available but are marketed chiefly in Asia [36]. Further developments have allowed the list of approved therapeutic applications of BoNT to constantly expand beyond strabismus and dystonia, with over 12 different medical conditions now approved in 2022, and a growing cosmetic market for BoNT treatments [13,14,37].

## Classification of *C. botulinum* strains and neurotoxins – is a species a species?

**Figure 1.**
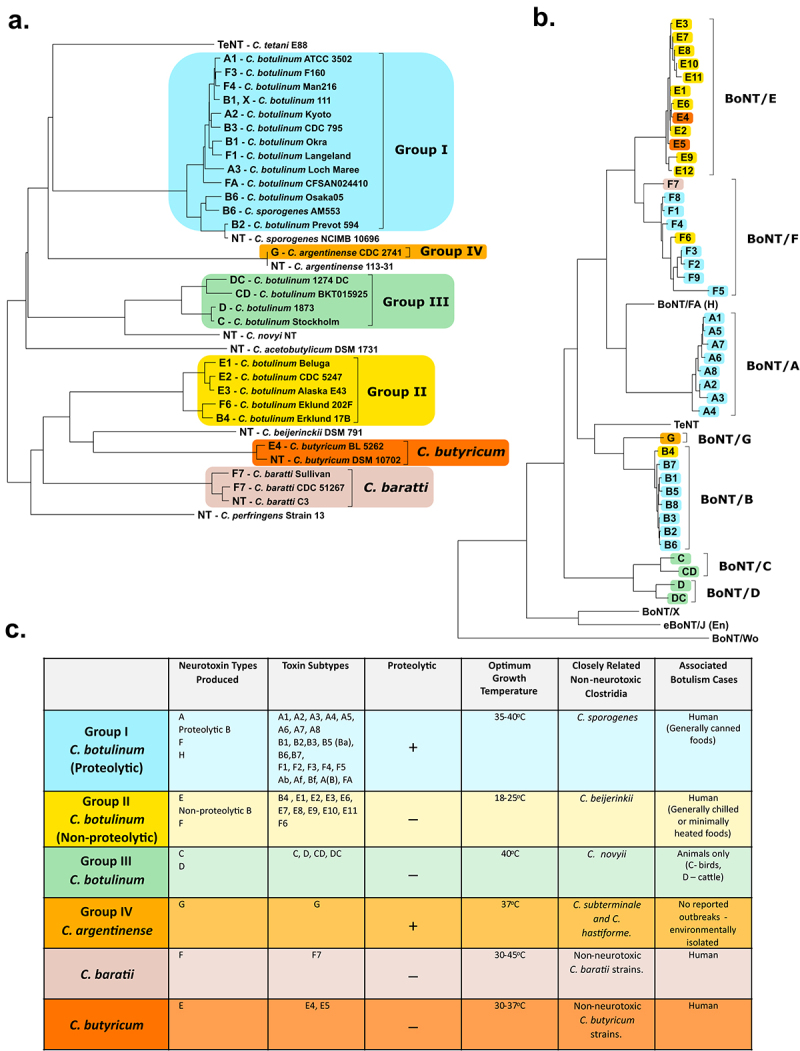
Classification of *C.* *botulinum* and the other BoNT producing strains. **(a)** A phylogenetic dendrogram of a representative selection of strains producing the seven different serotypes of BoNT and other related clostridia (NT – nontoxigenic strain). Constructed by average nucleotide identify from published whole genome assembly sequences and neighbour-joining tree method **(b)** Dendrogram comparing the protein sequences of all BoNT toxin types including the tetanus toxin (TeNT). Amino Acid sequences analysed by ClustalW alignment and tree created using the Maximum Likelihood method. **(c)** Table summarising the *Clostridium* spp. known to produce the BoNT; depicting the six phylogenetically distinct BoNT neurotoxic clostridia. Colours throughout refer to the group the strain or toxin type belongs to. All evolutionary analyses were conducted in MEGA11, with prior multiple sequence alignment of whole genomes by CLC Genomics Workbench 21.0.3 (CLC Bio, Aarhus, Denmark).

The species of *C. botulinum* historically encompassed all bacteria that produced a BoNT, this single criterion for inclusion has led it to be a highly diverse species of bacteria [38,39]. This taxonomy was adopted in 1950s to prevent confusion among clinicians and scientists when handling or diagnosing the dangerous bacterium. Within the species, four phylogenetically distinct lineages are apparent, known as *C. botulinum* Groups I-IV, collated based on genetic heterogeneity and physiological factors [40,41]. Greater similarity can be seen to other clostridial species than between the groups of *C. botulinum*, the only common denominator in the groups being the production of a BoNT [42]. This diversity can be seen in Figure 1.

Group I and II strains are most commonly associated to human botulism cases, whereas Group III strains are responsible for avian and some mammalian animal cases. Group IV strains, renamed to *Clostridium argentinense*, are rarely associated to illness and produce BoNT/G [43]. Neurotoxigenic strains of *Clostridium sporogenes* (classified in Group I), *Clostridium baratii* (often referred to as Group V) and *Clostridium butyricum* (often referred to as Group VI) are also known to produce BoNT/B, BoNT/F and BoNT/E neurotoxins, respectively [40].

The BoNTs have traditionally been divided into seven toxin serotypes from A to G, with subtype variants designated within each toxin type, labelled by a sequential number in order of discovery [41]. In 2016, a novel serotype was discovered in an existing *C. botulinum* strain, strain 111 which also produces BoNT/B2 neurotoxin, by whole genome and bioinformatic approaches. This serotype was named BoNT/X and although the toxin was proved to cleave a range of SNARE targets it is unknown if the BoNT/X gene cluster is expressed natively [44]. A chimeric toxin, BoNT/FA, was briefly thought to be a new BoNT/H serotype until further analysis uncovered it to be a chimeric toxin [45–47]. Toxin serotypes are categorized based on the toxin’s ability to be neutralized by monoclonal antibodies, while subtypes tend to be classified through differences in amino acid sequences, although subtypes can vary by 2.6–31.6% at the amino acid sequence level [42,48,49]. By this criterion, only four serotypes have designated sub-serotypes; BoNT/A, BoNT/B, BoNT/E and BoNT/F.

Strains from different groups can produce the same toxin serotype (e.g. BoNT/F is produced by Groups I, II and V) and subtypes of the same serotype can vary in biological activity [50]. Most characterized strains produce only a single serotype of neurotoxin, but some have been observed to produce two different serotype toxins, termed bivalent strains. A nomenclature for such strains has been devised where; (i) AB expresses equivalent amounts of each encoded toxin (ii) Ab, Af, Ba, and Bf predominantly express the capitalized major toxin and less of the lower case minor toxin (iii) A(B) called “A silent B” where a truncated BoNT/B produces no active toxin [51]. Additionally, mosaic toxins sometimes known as chimeric toxins, naturally exist which are a single BoNT composed of domains derived from different serotypes, such as BoNT/CD, BoNT/DC, and BoNT/FA. For instance, the BoNT/DC toxin has a C-terminal heavy chain similar to that of BoNT/C toxins but a light chain and an N-terminal heavy chain almost identical to BoNT/D [52–54]. Group III strains can additionally produce a non-neurotoxic binary toxin known as C2, a cytotoxic actin ADP-ribosylating toxin, and an C3 exoenzyme which has led to BoNT/C confusingly sometimes referred to as the C1 toxin [55,56].

The harbouring of the toxin gene clusters on plasmids and bacteriophages, as well as the presence of mobile genetic elements immediately flanking the toxin genes in several species, indicates horizontal gene transfer has played a role in acquisition of the cluster and explains the large diversity of BoNT-producing isolates identified [42]. Through the use of whole-genome sequencing and bioinformatics methods, in 2015 the first non-clostridial BoNT homologue was discovered in *Weissella oryzae* SG25 (BoNT/Wo) able to cleave VAMP2, and later in 2018 a second was found in *Enterococcus sp. 3G1_DIV0629*, a close relative of *E. faecium*, and termed eBoNT/J, with the ability to cleave VAMP 2 and SNAP 25 [57–59]. A more distantly related BoNT homolog in the genome of *Chryseobacterium piperi* (BoNT/Cp1) was discovered, but was designated a BoNT-like toxin, clustering outside the BoNT family of toxins with an approximately 15% overall sequence identity [60]. These discoveries challenge the established dogma that BoNTs are exclusively clostridial toxins. Of particular importance are the BoNT/A1 strains since they induce the most severe, longer-lasting forms of human botulism, and for that reason this specific toxin is predominantly selected for therapeutics and aesthetics [61,62].

With each *C. botulinum* Group generally considered to be its own distinct species, it has been proposed, although currently not widely adopted by the field, that the Group names I-IV be replaced using the old Latin binomial nomenclature. To differentiate the proteolytic from the nonproteolytic neurotoxin-producing clostridia, the proteolytic *C. botulinum* Group I species could be known as *Clostridium parabotulinum*, whilst the nonproteolytic Group II strains remain as *Clostridium botulinum* [63]. The Group III BoNT/C and BoNT/D producing bacteria, due to the similarities to *Clostridium novyi*, are proposed to be called *“C. novyi sensu lato”* [64]. Whilst Group IV, *C. argentinense*, preserves its current species name, as should the other BoNT-producing clostridia [65,66].

## Botulinum and bioterrorism

The use of biological agents for bioterrorism or bio-crime purposes continues to be of global concern [67,68]. BoNT is classified as among the highest risk threats, being listed as a Category A agent by the CDC in the US and a Schedule 5 biological agent in the United Kingdom [69,70]. Due to its potential to be utilized as a biological weapon, the storage and handling of BoNT neurotoxic strains requires enhanced security arrangements and regular audits. BoNT is one of the most potent biological agents having the lowest lethal dose of any natural substance known [71,72]. The human lethal dose (LD_50_) is dependent on the route of entry into the body, but assuming an average body weight of 70 kg, 70 µg of ingested, 0.7–0.9 µg of inhaled and 0.09–0.15 µg of intravenously administered concentrated BoNT/A toxin would be lethal [73].

Historically, there have been several examples of state-sponsored BoNT production for use as a biological weapon [74]. Under the leadership of Saddam Hussein, an extensive biological weapons program was overseen in Iraq between 1985 and 1991, where 19,000 L of concentrated BoNT was stockpiled for weaponization [75]. This would have been enough to kill the entire human population three times over. An aerosolized release of purified toxin is a scenario of concern to counter-terrorism organizations, however, BoNT is rather unstable in the environment, and would degrade within a couple of hours of release [76]. The most notorious report of BoNT bioterrorism is by the Japanese religious cult, Aum Shinrikyo, who failed to successfully release aerosolized BoNT preparations on three separated occasions in Tokyo prior to the group releasing Sarin nerve gas on the Tokyo subway in March 1995 [73].

Another scenario of concern is a deliberate contamination of beverages with BoNT. In liquid preparations, BoNT has been described as being comparatively stable, with 50% residual toxicity after 70 days [77]. An intentional release of BoNT into the water supply in the developed world would be prevented by routine chloride treatment destroying the toxin [78]. The tasteless, colorless and odorless nature of BoNT in solution, alongside the short incubation times that leads to a potentially fatal condition, makes a BoNT-based attack through injection or lacing of foods a worrying scenario [79]. Close monitoring of botulism cases, development of suitable diagnostics and improvement of treatments remains crucial.

## Botulism – a neuroparalytic disease

Botulism is a serious and potentially fatal neuroparalytic disease of humans and animals, in which the sole causative agent is the BoNT. Human botulism is characterized as a bilateral descending muscle weakness, symptoms generally begin in the cranial nerves presenting as blurred or double vision, dry mouth, and difficulty speaking [3]. The classical early physical presentation of botulism can be remembered using the “four D’s” mnemonic: dysarthria, diplopia and dysphonia, dysphagia. As the disease progresses to the extremities of the body with the dissemination of the neurotoxin, weakness to the muscles of the torso and upper limbs occurs. If left untreated, the respiratory muscle groups are affected, and death is caused by respiratory failure [80].

Despite the extraordinary potency, of BoNT cases of human botulism are now relatively rare with only an average of 160 reported cases of botulism annually in the United States according to CDC data [81]. Deaths due to botulism are now also rare, over the last century the fatality rate of human botulism cases dramatically decreased from 70% to currently less than 5% in the developed world [82–84]. This drop is primarily due to improved clinical recognition, treatment, and rigorous food processing measures. In animals, however, botulism is still prevalent in both wild and domesticated communities, in which deadly outbreaks occur through natural cycles of environmental transmission [85].

**Figure 2.**
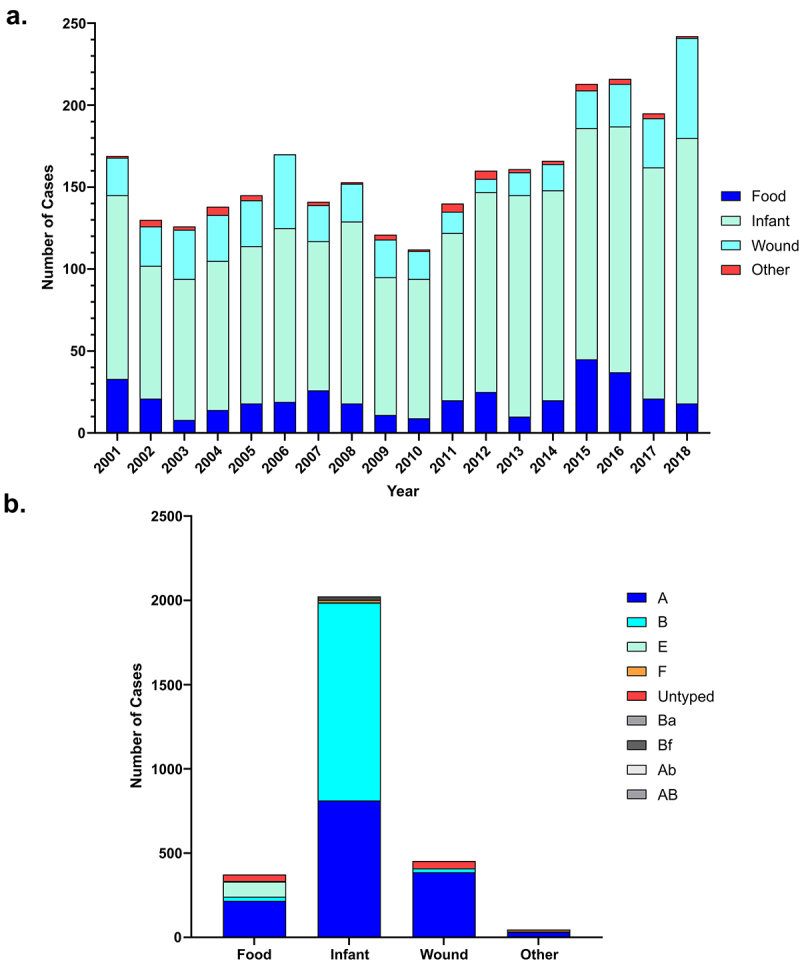
Reported botulism cases in the U.S. from 2001–2018. (a) Graph depicts the number of cases of each type of botulism, demonstrating the prevalence of infant botulism compared to other clinical types. (b) Shows the causative toxin type of reported cases if specified. All data obtained from the CDC national botulism surveillance annual summaries [110].

In humans, the disease is classified into three main clinical types dependent on the route of entry into the body; foodborne, wound, and infant botulism. The prevalence of each can be seen in Figure 2. Symptoms across all forms of botulism are broadly uniform, with the exception that foodborne cases may include additional gastrointestinal symptoms of diarrhoea, nausea, vomiting, and abdominal cramps prior to neurological symptoms [86].

### Foodborne botulism

Foodborne botulism is caused by the ingestion of food contaminated with preformed BoNT, with as little as a few milligrams of contaminated food enough to cause symptoms and perhaps death if left untreated [10]. Symptoms typically present 12–72-h post-consumption of the contaminated food, dependent on the quantity ingested [73]. Foodborne botulism was historically the most prevalent form of human botulism and is usually associated with uncooked foods or the inadequate processing of food stuffs both commercially and when home prepared. Preservation techniques such as fermentation or pickling, followed by canning or bottling of foods without prior heat treatments pose the greatest threat as they create the anaerobic conditions required for spore germination and bacterial growth [3]. The introduction of certain food control measures; the “botulinum cook” (121 °C heat treatment for 3 min), freezing, and refrigeration (below 4 °C), when applied correctly, have greatly decreased foodborne outbreaks this century [87–90].

Vegetative bacterial growth and the generation of toxin occurs only under anaerobic, low salt (salt concentration >5% throughout food), high water activity, and non-acidic environments (pH < 4.6) [91–93]. Bearing this in mind, the product formulation can help prevent incidences. BoNT is temperature sensitive, and any preformed toxin in the food stuffs can be inactivated by heating at 85 °C for at least 5 min [78]. The spores can only be inactivated by the “botulinum cook,” although temperature can be reduced in the presence of increased pressure [10,87,94].

Group I and II *C. botulinum* are associated with outbreaks of human foodborne botulism. BoNT/A and BoNT/B toxin serotypes are the most prevalent, with cases of BoNT/E and BoNT/F being rarely reported (see Figure 2) [91]. Proteolytic, Group I *C. botulinum* strains are usually associated with outbreaks linked to shelf-stable commercially processed canned foods due to their high spore heat resistance [91,92]. Group I strains are mesophilic and as such have a minimum growth temperature of 12 °C [10]. However, non-proteolytic Group II strains are able to grow at temperatures as low as 3 °C are more commonly associated with minimally heated or chilled food cases, such as fish or marine products [92,95]. When an outbreak does occur, it can have significant commercial implications for the food and drink companies involved. In a recent outbreak in the US, it was found that inadequate refrigeration and/or heat treatment of cans of chili sauce for hot dogs resulted in the product being compromised, necessitating the recall of 111 million cans and ultimately leading to the closure of the canning company [96].

Food-borne botulism remains a prevalent disease in domesticated and wild animals, where outbreaks among farm livestock and wetland birds tend to spread rapidly, which can lead to devastating epidemics due to the favorable cycles of transmission in the environment [97]. *C. botulinum* bacterial growth in anaerobic niches of decaying organic matter leads to production BoNT, which is ingested by animals, birds, or fish leading to death. Death can either be directly from a lethal dose of neurotoxin or consumption of a non-lethal quantity that decreases the animal’s fitness, causing it to become prey or outcompeted for resources. This contaminated carcass can then propagate further infections to other animals that feed on it [7,98]. This infection cycle can be further enhanced through neurotoxin ingestion by BoNT-insensitive insects and other invertebrates, such as maggots, worms or shellfish and subsequent consumption by a toxin sensitive animal [99]. Animal outbreaks tend to be associated with BoNT/C and BoNT/D toxin serotypes, although interestingly equine botulism is more commonly related to BoNT/B [100].

### Infant botulism

Environmental *C. botulinum* spores routinely pass through the human digestive system with no ill effect as the healthy, mature human gut is not conducive to clostridial spore germination [3]. However, an infant’s immature gastrointestinal tract (typically between 1 and 6 months old but reported in babies up to 12 months of age) is more amenable for *C. botulinum* colonization following the ingestion and germination of spores [101]. This is due to immature gut physiology and/or underdeveloped gut microbiota. This results in the endogenous production of the neurotoxin by the bacteria, absorption from the bowel lumen, and so-called “floppy babies” with an inability to swallow or suck, with symptoms typically beginning 18 to 36-h post-ingestion [102]. The majority of cases arise from consumption of untreated natural food stuffs, honey, and corn syrup, for example, are frequently found to contain a high concentration of spores [103–105]. Other sources of spores have originated from contaminated powdered baby formula and household dust [106,107]. Infant botulism was first reported in 1976 and this century it has been the predominant form of human botulism cases in the US, as can be seen in Figure 2 [108,109].

### Wound botulism

Wound botulism was first formally reported in 1951 and is caused by the absorption of BoNT into the blood stream following contamination of a wound with *C. botulinum* spores from the environment [111]. The anaerobic conditions within a wound abscess provide the environment needed for spore germination and colonization. Due to the nature of infection, the presentation of clinical symptoms typically takes longer than those of other clinical forms of botulism, typically presenting between 4 and 14 days [112]. Historically, cases were comparatively rare and usually implicated with trauma events, however the increasing prevalence of injecting drug-users led to a dramatic increase in the number of wound botulism cases [113–115]. In particular, an epidemic of cases in the state of California in the United States arose due to increased usage of black tar heroin through the late 1980s into 1990s [116,117].

### Less prevalent forms of botulism

Rarely cases of botulism arise that do not fit into the three main forms and are caused by much rarer manifestations of the condition. Adult intestinal toxemia botulism is caused when adults, like infants, suffer from *C. botulinum* colonization of the gastrointestinal tract due to depletion of the natural gut microbiota. This can arise following heavy antimicrobial use, gastrointestinal surgery, or inflammatory bowel disease [118–120]. Rare botulism cases also occur due to the incorrect administration of concentrated BoNT for therapeutic and cosmetic purposes, called latrogenic botulism [121–123]. An extremely rare non-natural form of intoxication following the absorption of BoNT across the respiratory mucosal membranes has been termed inhalational botulism [124,125]. This mechanism of transmission is generally discussed when hypothesizing the potential for a bioterrorism event utilizing aerosolised BoNT [126]. No person-to-person transmission of botulism has ever been reported.

## Treatment of botulism

The present treatment for botulism relies on symptomatic control, mechanical respiratory support, and the passive intravenous administration of botulinum antitoxin (BAT). It is possible to survive botulinum intoxication without antitoxin therapy through modern patient supportive care, such as mechanical ventilators, but recovery and the duration of hospitalization will be prolonged and costly [127,128]. The current antitoxin is a heptavalent polyclonal antibody formulation (HBAT®) which contains neutralizing antibodies against seven toxin serotypes; A, B, C, D, E, F, and G and has superseded the older trivalent antitoxin in US and UK, which is only able to neutralize serotypes A, B, and E [129,130]. Some trivalent antitoxins are still used in several European countries [131]. BAT is derived from equine sources, by blending sterile sera obtained from horses hyperimmunized with *C. botulinum* toxoid strains for each serotype. It has been recently proposed that safer recombinant subunit fragments of the toxins can effectively trigger the equine immune response and lead to production of BAT with a similar high efficacy against the neurotoxins [132]. HBAT® was approved by the FDA in the US in March 2013 but is used unlicensed in the UK [133].

Serum-based antitoxin treatment has numerous limitations, not least its inability to neutralize toxin already in the intracellular environment. The antitoxin can only bind and neutralize toxin in the bloodstream, through blocking binding of the heavy-chain receptor to the presynaptic receptors, resulting in the removal of the toxin-antitoxin complex from circulation [134]. Consequently, the antitoxin should ideally be administered as quickly as possible after the onset of symptoms, to minimize the amount of toxin internalized [135]. Data suggests that patients treated with HBAT® within 48 h of the onset of symptoms require fewer days in hospital and require less intense care [127,129]. Hence, clinicians are advised that all cases of suspected botulism should be treated without delay and not to await laboratory diagnosis due to the fast-irreversible progression of intoxication [128].

As BAT is an animal-derived from antibody preparation, and as such is foreign to the human body there is a risk of serious hypersensitivity side effects, such as serum sickness and anaphylaxis [136,137]. Anaphylaxis occurs in less than 2% of the treated patients but incidence of serum sickness and other milder allergic reactions is less well documented [138]. Considering these complications sometimes seen with equine antitoxin treatment in adults, a bivalent human-derived immunoglobulin preparation (BIG-IV) has been used to treat infant botulism since the FDA in the US approved the use of BabyBiG® in 2003 and outside of the US since 2005 [139–141]. BIG-IV is a purified immunoglobulin preparation against serotypes A and B only, derived from adults immunized with recombinant *C. botulinum* vaccine strains, and as such has a larger half-life with respect to equine antitoxin [139]. BabyBIG is effective and despite its high cost per treatment, $45,300 per vial as of 2017, it was predicted to have saved >$85 million in hospital charges in the US in its first 12 years post-licensure in 2003. It also reduced the average length of hospital stay by 3.6 weeks per infant treated compared to the placebo-treated patients in the 1992–1997 BIG-IV pivotal clinical trial [142]. Despite these benefits, availability of BIG-IV is limited in some countries due to high upfront costs when compared to traditional equine antitoxin (Vanella de Cuetos et al. 2011).

For the treatment of wound botulism cases, the antitoxin is administered followed by reduction of the bacterial load by antibiotic treatment (penicillin and metronidazole) and surgical debridement of the infected wound abscess to prevent further toxin production [143]. This is in sharp contrast to infant and adult intestinal botulism cases where antibiotic treatment is not recommended due to possible worsening of neurological symptoms following further gut dysbiosis and potential lysis of vegetative *C. botulinum* cells resulting in the release of intracellular neurotoxin [141,144,145]. In cases of foodborne botulism gastrointestinal decontamination may be considered, such as gastric lavage or induced emesis, only in cases were the ingestion of contaminated meal was recent [131].

Supply of antitoxin is limited due to the long immunization course required before adequate levels of antibodies are developed in the sera of the equine production host and the short half life of the administered immunoglobins in circulation requires large quantities of the antitoxin per dose [146–148]. In view of the shortfall of serum-based antitoxins, alternative therapeutics for botulism poisonings have been explored. Several human monoclonal antibody therapeutics to BoNTs are currently undergoing clinical trials, following demonstration of prior efficacy and safety [149–153] and single-domain antibodies (VHH) have also shown promise [154,155]. Antibodies by nature are short lived however, and therefore do not persist in the human body. Recently, a recombinant adeno-associated virus expressing a BoNT/A antibody has been reported to give long-term protection in animals to an otherwise lethal dose of toxin [156].

The threat of BoNT being utilized as a biological weapon has led to the exploration into several vaccines to provide protection against the effects of BoNTs, allowing clearance of the toxin from the circulation before it can bind to nerve endings and cause symptoms [157,158]. The current rarity of the disease, combined with the fact that vaccination precludes the individual from the potential therapeutic benefits of BoNT, means that population-wide immunization programs have not been established. A pentavalent (ABCDE) formalin-inactivated toxoid vaccine against botulism was used for nearly 50 years in a program to vaccinate workers at high occupational risk of exposure to botulinum toxins, such as researchers and manufacturers in the US [159,160]. This program ended in November 2011 due to the expiry of the initial batch of vaccines and reduced efficacy of the toxoid vaccine [161]. Since then sera, protein-based, recombinant viral vectors and DNA vaccines are currently being explored as replacements [147,162].

Dependent on the quantity and serotype of the causative BoNT, recovery from the condition can range from a few weeks to several months. With prompt treatment and administration of the antitoxin, a full recovery can occur in as little as two weeks with no lasting symptoms [128,163]. Recovery from intoxication and restoration of neurotransmitter release is a consequence of the natural protein turnover causing degradation of the BoNT in the neuronal termini and the regeneration of nerve endings from the intoxicated neuron [164–167]. The alterative toxicity and persistence of toxin serotypes is thought to be associated with the resistance of the proteins to ubiquitin-proteasome system (UPS) mediated protein turnover [168,169]. Treatments to accelerate botulism recovery times by increasing internalized BoNT turnover are being explored [170].

## Diagnostics for botulism and BoNT

For the successful treatment of botulism, a rapid diagnosis of the condition is essential to dictate treatment and prevent fatal consequences. When diagnosing botulism, in addition to the observation of clinical symptoms and patient history, physicians should seek a positive laboratory diagnosis. Symptoms of other neurological conditions, such as stroke, Guillain-Barré syndrome, myasthenia gravis, tick paralysis, and Eaton-Lambert syndrome, may appear similar to those of botulism, especially in the early stages of intoxication [86,171–173]. However, treatment should not be excessively delayed in anticipation of laboratory test confirmation, due to the increased morbidity associated with delay in positive cases [128]. Differential diagnosis is more commonly associated with sporadic or unconnected cases, whereas the observation of several linked cases often helps make a botulism diagnosis more apparent.

A confirmed diagnosis can be made if biologically active BoNT is found in consumed food, debrided wound tissue, fecal matter, or serum of the patient. The mouse lethality bioassay is the current gold standard for BoNT detection, which involves injection of the patient sample into laboratory mice [128,174]. If BoNT is present, the mice will develop classical symptoms of botulism typically within 24–48-h post-injection dependent of concentration of neurotoxin present [175]. The toxin type of the causative BoNT can be identified through administration of specific antitoxins to the infected mice. Although *in vivo* mouse bioassays are extremely sensitive (lower detection limits in the range of 5–10 pg/mL of toxin) and versatile in sample types that can be tested, they are relatively expensive, can take several days requiring specialized facilities and trained personnel, and due to the use of animals are ethically unfavourable [176]. In 2019, it was estimated that approximately 400,000 animals are used for industrial BoNT batch testing per year in Europe alone [177].

Cases can also be confirmed following detection of BoNT-producing bacterial species in the stool or wound tissue of suspected individuals. This can be confirmed by polymerase-chain reaction (PCR) tests to detect the presence of the toxin genes DNA (*bont/*A-G) in samples [178–181]. Results can be provided within hours and are generally satisfactory for diagnosis but cannot definitively confirm the presence of the causative proteinaceous toxin. A laboratory diagnosis provides evidence to identify and isolate the suspected food source [176,182].

For successful laboratory confirmation specimens must be collected as soon as a botulism case is suspected, as toxin levels swiftly decrease in serum and stool samples over time as it is internalized into neurons. A delay in sample collection, or storage above refrigerator temperature (2 °C − 8 °C), can lead to false-negative results. Serum samples must also be collected prior to BAT administration, but stool samples may be collected post-treatment as *Clostridium* species are not affected by the antitoxin, but prior to antibiotic therapy [128].

For some years, an *in vitro* replacement for the mouse bioassay has been pursued that would provide; (i) accelerated results within hours, (ii) an equivalent or higher level of sensitivity, (iii) a more cost-effective solution per test, (iv) better accessibility both in terms of ease of use and in the equipment and facilities required and, (v) effective detection in various matrices. Additionally, a candidate would ideally be able to accurately detect multiple serotypes of active toxin simultaneously. An *in vitro* assay would be beneficial not only for patient diagnosis, but for industrial food safety and environmental surveillance. The advancement of alternative BoNT detection methods has been an active area of research over recent years [183–187].

Pharmaceutical companies producing BoNT preparations for therapeutic or cosmetic applications are currently leading the way in the development of alternatives to the mouse lethality assay. Several have developed the FDA and EU approved cell-based assays (CBAs) to dramatically reduce their use of animal testing to detect and test BoNT potency [177]. Allergan was the first to develop a BoNT CBA, which was approved by the FDA in 2011 (and in the EU in 2012) and was outlined in a publication in 2012 [188]. Merz and Ipsen later developed their own CBAs, with EU approval granted in 2015 and 2018, respectively [189,190]. A limitation of these assays is that they were developed for potency testing of purified single serotypes and are not suitable for clinical diagnostics or food testing with complex sample types [191].

In a detailed review on the advances in BoNT diagnostic methods by Hobbs *et al*. in 2019, numerous exciting technologies were discussed, highlighting a centrifugal microfluidic assay, a colorimetric assay, an SPR-based biosensor, and the EIS (Electrochemical Impedance Spectroscopy) platform as particularly promising based on sensitivity, speed, and cost [192–196]. Each require further validation and optimizations before they could be considered as an industry-wide replacement for the standard mouse bioassay.

## The causative agent – the botulinum neurotoxin

**Figure 3.**
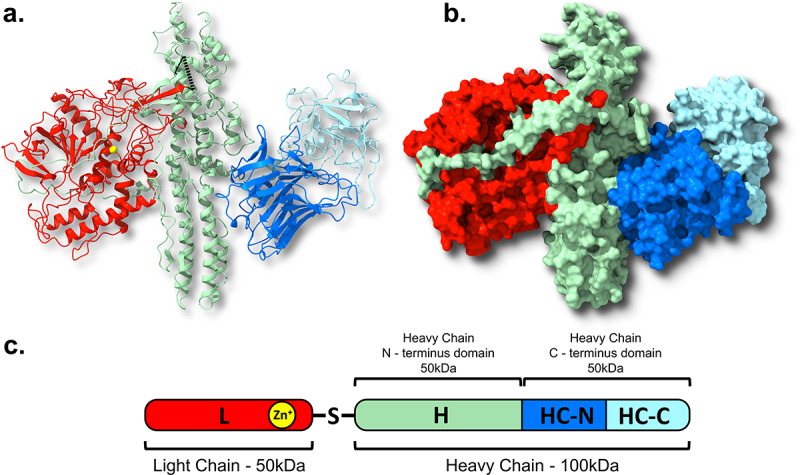
The classical structure of the BoNTs. Depicted is the BoNT/A1 from c. botulinum ATCC 3502. The bontA1 gene is translated into a single 1295 amino acid polypeptide (c.), which is cleaved into a light chain (L – coloured red) and a heavy chain joined by a disulphide bridge between Cys430 and Cys454 residues (shown in black). The heavy chain contains an N-terminal translocation domain (HN – coloured light green) and a C-terminal receptor binding domain, which is formed of two subunits (HC-N and HC-C coloured dark and light blue respectively). The light chain is the catalytic domain and consists of a zinc metalloprotease (the Zn^2+^ atom shown in yellow). The light chain is encircled by a hydrophobic peptide belt domain of the HN (coloured in dark green in c.). Crystal structures were prepared using UCSF ChimeraX (PBD ID: 3BTA) with a. displayed in ribbon format and b. a spacefill representation [198,202].

BoNTs are encoded by *bont* genes, which are located either on chromosomal, plasmid, or phage elements [197]. Common to all serotypes, the toxin is initially translated as a 150 kDa single-chain peptide [198–200]. This precursor then undergoes a post-translational cleavage losing 11 amino acids in a process termed nicking, which occurs 50 kDa from the N-terminal end of the polypeptide [201]. The mature and active toxin (known as the di-chain holoenzyme) is formed of two chains, a 100 kDa heavy chain and 50 kDa light chain, linked by non-covalent interactions and a single interchain disulphide bridge (Figure 3) [203,204]. The light chain incorporates the catalytic zinc-dependent metalloprotease activity, a zinc atom bound to a HEXXH motif, sheltered within the core of the structure [205,206]. The heavy chain is composed of two domains; the N-terminal translocation domain (HN) and the C-terminal receptor-binding domain (HC). The HC is further broken down into two subunits (HC-N and HC-C) with the interface between the subunits providing the space required for the glycosylated receptors (gSV2C) of the synaptic terminal to bind [207]. The HC-N is responsible for interacting with membrane lipids, while the HC-C subunit is involved in nerve cell binding and is crucial regarding the internalization into, and trafficking within the nerve cell [208–210]. The HN translocation domain includes a peptide belt that wraps around the light chain and facilitates translocation of this catalytic domain from the vesicles into the synaptic cytosol. It is also thought that this belt functions to partially occlude the active site, preventing any catalytic activity of the light chain prior to separation from the heavy chain [211–213].

**Figure 4.**
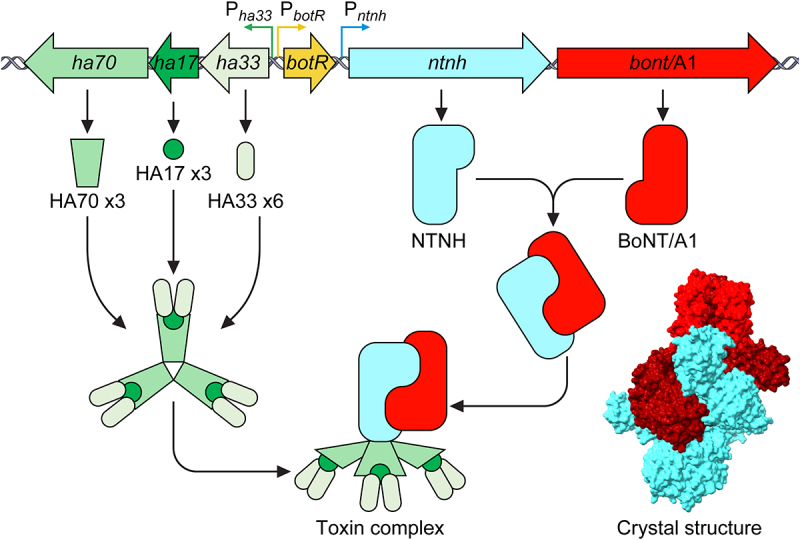
Structure of the progenitor complex (PC) and BoNT-NTNH heterodimer. The NTNH and BoNT interlock to form a heterodimer with the catalytic light chain positioned on the exterior, held in place by the peptide belt of the heavy chain translocation domain (HN). The HA proteins assemble to form a symmetric tripod structure (B.). The NTNH interacts with the three HA70 proteins of each “arm” to complete the PC. Crystal structure of BoNT/A1- NTNH/A1 is shown in the bottom right and was prepared with UCSF ChimeraX with the BoNT heavy chain coloured dark red (PDB ID:3VOA) [202,216].

The mature BoNT forms a progenitor complex (PC) with one or more neurotoxin associated proteins (NAPs) through non-covalent interactions [214]. In all serotypes, the BoNT forms an interlocking heterodimer with the non-toxic non-hemagglutinin (NTNH) protein, which is structurally similar to the toxin but lacking the zinc-binding motif characteristic of the clostridial neurotoxin metalloproteases [215,216] (Figure 4). The BoNT-NTNH dimer complex associates with a number of other NAPs. In some strains, the NAPs are hemagglutinin proteins (HA) and in others OrfX proteins (OrfX1, OrfX2, OrfX3, and P47) [42]. The OrfX proteins are found in BoNT/E, BoNT/F and some BoNT/A serotypes such as BoNT/A2. The HAs are found in BoNT/B, BoNT/C, BoNT/D, BoNT/G, and all BoNT/A1 serotypes, although one HA^−^ OrfX^+^ A1 strain has been described [42,217].

In most BoNT/A1 strains, including the model strain ATCC 3502 which was the first *C. botulinum* to have its genome sequenced in 2007, the progenitor complex is formed of three HA proteins named; HA33, HA17, and HA70, the number referring to the protein size in kilodaltons (alternatively named HA1, HA2, and HA3, respectively) [217,218]. The PC is thought to be approximately 760 kDa in a 1:1:3:3:6 stoichiometric ratio (BoNT:NTNH:HA17:HA70:HA33). The HAs form a trigonal three-armed structure on which the BoNT-A1/NTNH-A1 complex sits, to make a “phage-like” structure (Figure 4) [219]. This structure suggests that the NTNH protects the toxin from chemical or enzymatic attack, such as low pH conditions and proteases associated with oral ingestion or present within decaying biological matter [15,216]. Conversely, the HA complex is believed to form a docking station on the intestinal lumen, as they contain multiple-carbohydrate binding sites, to allow the efficient transport of the BoNT across the intestinal epithelium into the bloodstream [220–223]. Additionally, the HA proteins are known to disrupt the intestinal epithelial barrier through binding of E-cadherin between epithelial cells, compromising the integrity of the epithelium and facilitating absorption of the toxin increasing oral toxicity [224–227]. The PC structures formed by the Orf proteins remain unknown but are thought to perform a similar function to the HA proteins, having been shown to be involved in lipid binding [228,229]. In culture, BoNT/A1 PCs have been found to exist in three different forms; a 12S M-complex (~300 kDa; BoNT and NTNH only), 16S L-complex (~760 kDa; BoNT, NTNH, HA17, HA33, and HA70) and 19S LL-complex (~900 kDa; a dimer of 16S L-PCs). The 19S complexes are thought to only occur in high concentration [219,230].

### Mechanism of action

**Figure 5.**
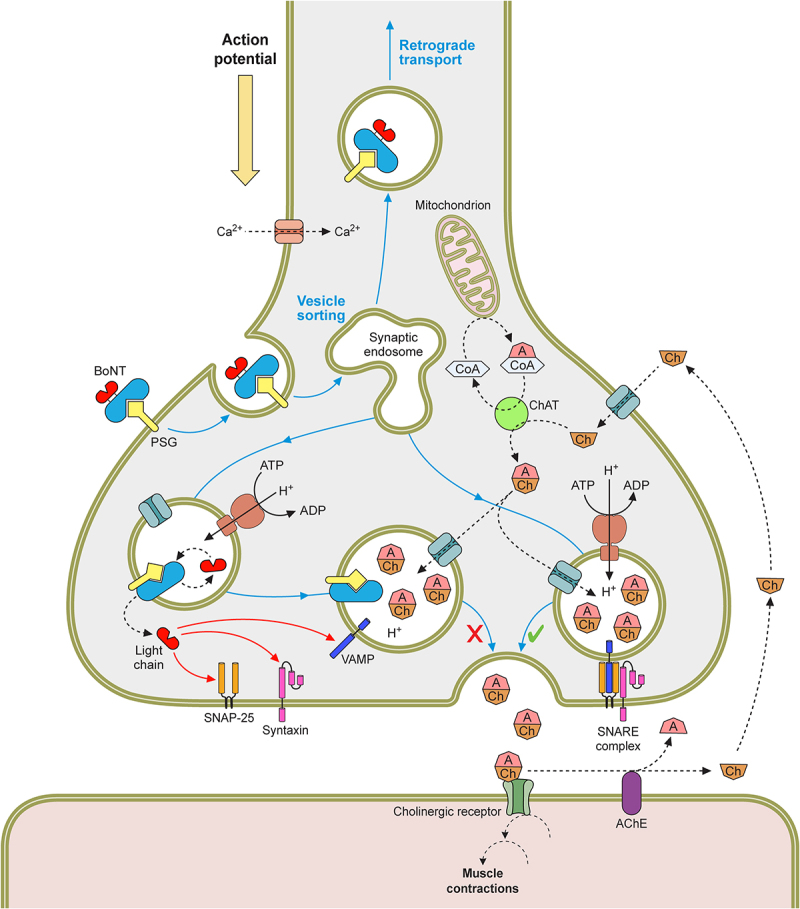
The mechanism of BoNTs at nerve endings. The signalling between the pre and postsynaptic cell is mediated by small molecules called neurotransmitters, at neuromuscular junctions (NMJ) this is acetylcholine. The neurotransmitter is stored in membrane bound synaptic vesicles inside the neuronal cytosol. Endocytosis of the empty vesicles triggers a V-ATPase proton pump to generate a pH gradient across the vesicular membrane, which drives the newly synthesised neurotransmitter molecules to enter the vesicles. The loaded vesicles bind to the interleaflet of the presynaptic membrane by the VAMP and synaptotagmin proteins in a process called docking. On docking, SNARE complexes form around the vesicle, and facilitate the fusing of the vesicle and the membrane in mechanism known as priming. As a result of a Ca2+ influx, caused by the depolarisation of the presynaptic nerve, SNARE proteins undergo a conformational change to allow the primed vesicle to release its neurotransmitter into the synaptic cleft. Through diffusion the neurotransmitter binds to receptors on the postsynaptic cell membrane, causing its excitation. This cycle is then repeated in readiness for future depolarisations. However, BoNT blocks neurotransmitter release from the presynaptic nerve terminal. The toxin is endocytosed along with empty vesicles and the light chain is translocated into the neuronal cytosol. This catalytic protein then cleavages SNARE proteins on the vesicular membrane or inner leaflet of the presynaptic membrane, dependent on the toxin serotype. This prevents vesicle docking and fusion to the presynaptic membrane and subsequently the release of neurotransmitter.

With the aid of the PC, the BoNT enters the blood circulation, via the lymphatic system if ingested, and disperses throughout the host tissues, localizing at peripheral nerve endings [231]. A dual receptor-binding approach to a polysialoganglioside (PSG) and a synaptic vesicle receptor (Syt or gSV2C) allows the BoNT to selectively target the presynaptic membrane of these nerves [232,233]. The receptor-bound toxin then enters the presynaptic cell via receptor mediated endocytosis (RME) [234]. The neurotransmitter refilling of the synaptic vesicles creates an acidic pH, causing a structural change of the heavy-chain translocation domain to form a pore-like structure, which allows translocation of the unfolded light chain from the vesicle to the neuronal cytosol [235–239]. In the cytosol, the interchain di-sulphide bond is reduced, releasing the catalytic light chain of the toxin, which subsequently folds to allow specific cleavage of their SNARE targets [240,241]. The SNARE proteins are part of the neurotransmitter exocytosis machinery, which allows fusion of the neurotransmitter vesicle and the neural membrane [242–244]. Cleavage of these SNARE proteins blocks neurotransmitter release into the neuromuscular junction, preventing postsynaptic excitation [245]. This results in the classical flaccid paralysis or muscle weakness observed in botulism cases. The overal mechanism of action is summarised in Figure 5.

**Figure 6.**
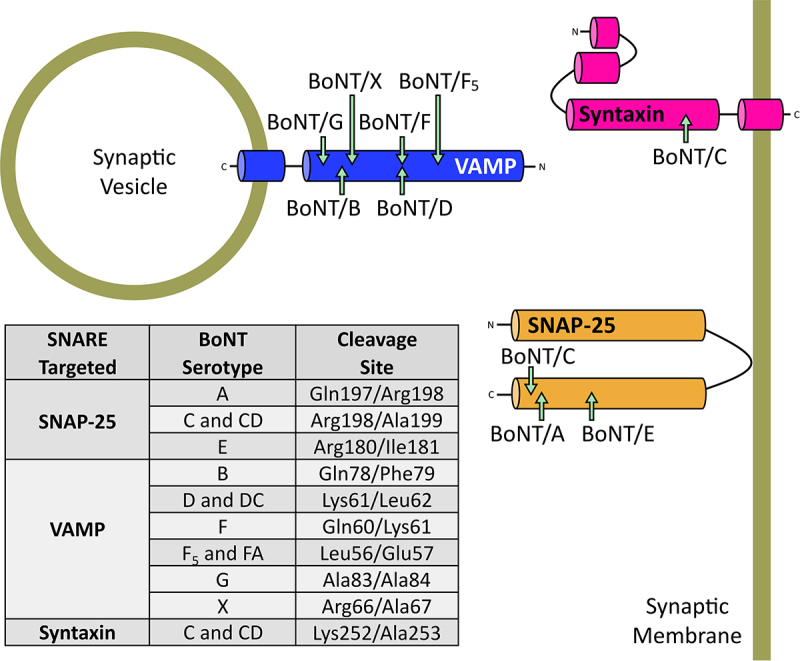
Alternative SNARE cleavage sites of BoNT serotypes. Specific residues cleaved in each of the SNARE targets and their respective BoNT serotypes are shown in the table.

The BoNT SNARE protein targets include the vesicle associated membrane protein (VAMP – overwise known as synaptobrevin), syntaxin, and the synaptosomal-associated protein of 25 kDa (SNAP-25) [246]. As the name suggests, VAMP is anchored on the outer membrane of the synaptic vesicle, whereas syntaxin and SNAP-25 are located on the inner leaflet of the neuronal membrane. Dependant on the serotype of the toxin, the light-chain metalloprotease cleaves the SNARE proteins at alternative peptide bonds, removing a segment of the cytosolic proteins and preventing the formation of the SNARE complex essential for neuronal vesicle docking and priming to the inner presynaptic membrane [245]. These alternative SNARE targets and cleavage sites are summarized in Figure 6.

Evidence strongly supports that the long duration of BoNT/A intoxication results from the retention of the toxin within the nerve termini [247]. However, some data suggests that short nature of the BoNT/A SNAP-25 truncation may play a contributing factor to its prolonged action [248]. The cleavage of SNAP-25 by BoNT/E, known to cause the shortest exocytosis blockade of only lasts 1–4 weeks in humans, removes a 26 residue segment from its C-terminus and does not allow for SNARE-complex formation [61,249]. Whereas in the case of BoNT/A serotypes, the cleavage of SNAP-25 removes only a small segment (9 residues) of the protein. This truncated SNAP-25 is still able to form a SNARE complex, yet this SNARE complex is non-functional, and is thought to give the cleaved protein a dominant negative effect, which enhances the persistence of BoNT/A function in neurons [247,250].

Historically, it was thought that BoNT exclusively acted at peripheral nerve terminals. Substantial evidence now suggests that some degree of retrograde transport occurs, similar to that of TeNT, which can give the toxin long-distance effects [251]. Axonal transport of BoNTs has been demonstrated in several studies, predominantly through the use of animal models, for instance where cleaved SNARE proteins were detected in neurons at least two synapses from the site of injection [252]. Evidence has also been presented in therapeutic studies in humans where BoNT activity has been observed distal to the site of peripheral muscular injection, e.g. through reduction in tibial nerve reflex following injection in the ankle plantar flexor [253,254]. Effects on the central nervous system (CNS) which have been observed following administration of BoNT can broadly be separated into two categories, indirect and direct. Many studies have demonstrated that BoNTs may indirectly affect the functional organization of the CNS through altered peripheral inputs, i.e. that the blockade of the neuromuscular junction (NMJ) has a knock-on effect, which may bring about plastic adaptive reorganization of the neuron.

Although the neurotoxin action at the CNS is not conclusive, there is increasing evidence of a direct effect of BoNTs on the CNS through retrograde transport, whereby following intramuscular injection, a small proportion of the BoNT travels into the axon rather than remain at the NMJ [255]. In one such study, the presence of cleaved SNAP25 in a mouse model has been observed through immunostaining in sections of hind paw, to sciatic nerve, dorsal root ganglia, and spinal cord, a strong indication for axonal transport of the BoNT along peripheral nerve to the spinal cord [256,257]. Although the application of these new findings to BoNT-based therapeutics targeting specific regions of the CNS is still in its infancy, it is an exciting and developing area, which may open up promising treatments for challenging neurodegenerative disorders.

### Neurotoxin gene regulation

**Figure 7.**
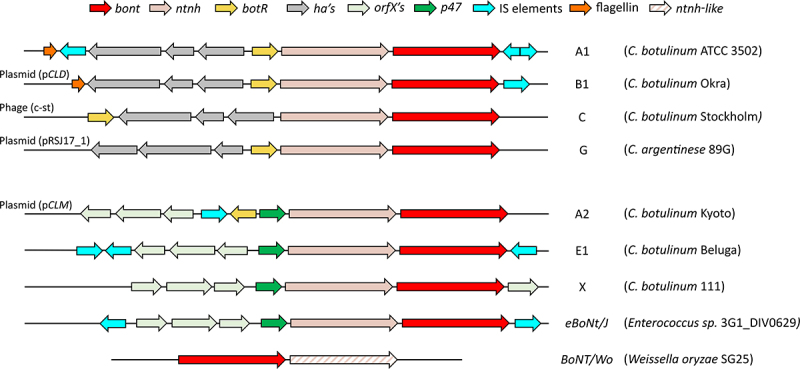
Botulinum neurotoxin gene cluster variation in a representative selection of neurotoxic strains. the diversity in the gene clusters and flanking regions, with ha clusters at the top and orfX at the bottom. The location of the gene cluster is indicated, if not chromosomal, on the left. The Group I strains either have the classical ha70-ha17-ha33-botR-ntnh-bont genes or the orfX3-orfX2-orfX1-botR-p47-ntnh-bont cluster. Group III are contained in ha clusters, although the alternative sigma factor gene, botR, is upstream of ha70. Group IV C. argentinese strains also have ha accessory proteins but the ha70 and ha33 genes are inverted relative to the conventional structure. Group II strains (with the exception of bont/B4 containing a ha70-ha17-ha33-botR-ntnh-bont cluster) have orfX3-orfX2-orfX1-p47-ntnh-bont clusters, which lack the botR gene completely; which is also observed in C. baratii F7 and the neurotoxic C. butyricum strains [267,268]. This diversity is likely attributable to recombination events and horizontal gene transfer between strains, which have also led to neurotoxin gene clusters present in non-Clostridium sp. such as Enterococcus sp. 3G1_DIV0629 and Weissella oryzae SG25 [59]. The Weissella neurotoxin gene is present without accessory genes, the ntnh-like gene downstream of bont/Wo lacks significant domains compared to the well conserved ntnh [269]. The insertion elements (IS) and flagellin genes flanking and internal to the gene clusters indicate the probable method of transfer into the strain as it evolved.

The genes encoding the botulinum neurotoxin and its non-toxic accessory proteins are always located proximal to each other, and this locus is termed the neurotoxin gene cluster. The location and overall composition of genes that comprise the cluster varies between strains, although the *ntnh* is always located upstream of the *bont* toxin gene and is co-expressed alongside the toxin in the same operon [258] (Figure 7). The conservation of the *ntnh* alongside the *bont* gene suggests they arose from a common ancestor, possibly through a gene duplication event [259,260]. The other accessory protein genes are generally located in a divergent polycistronic operon and consist of three genes grouped into two categories; the *orfX* genes or the *ha* genes. In addition to *orfX1, orfX2,orfX3*, an additional gene, *p47* (encoding a 47kDa protein), is always found in the cluster upstream of the *ntnh* [42]. There is no structural homology between the two sets of accessory proteins, and the function of the OrfX and P47 proteins is poorly understood. The role of OrfX-P47 may be similar to that of HA proteins in that they facilitate binding and transport of the PC into circulation through the intestinal barrier [223]. However, the fact that *orfX-p47* clusters have been found in numerous other unrelated bacteria and are frequently associated with other bacterial toxins suggests that their function is not exclusive to BoNT pathogenicity [261].

Located between these two divergent operons is the *botR* gene, previously termed *orf21*, which encodes a 21 kDa alternative sigma factor 70 (σ [70] that is responsible for the positive regulation of the entire toxin cluster, equivalent to *tetR* in *C. tetani* [262]. Binding sites for the BotR sigma factor are found in the promoters of cluster operons, binding directly to the core RNA polymerase enzyme, controlling the expression of the entire cluster. A BotR binding site is also found in the upstream promoter region of the *botR* gene itself, suggesting a positive regulatory loop of the entire gene cluster [263–265]. However, *botR* is not present in toxin clusters of BoNT/E producing strains, neurotoxic *C. baratii*, BoNT/X, and eBoNT/J strains, suggesting another form of regulation present in these cases [44,59,266].

In the *C. baratii* BoNT/F7 strain lacking *botR*, the promoters of the neurotoxin cluster were surprisingly similar to that of BotR regulated promoters. Immediately upstream of the neurotoxin gene cluster, a UviA-like regulatory protein was discovered with a similar DNA-binding-domain to that of σ [70] family, suggesting this UviA-like protein may act as an alternative sigma factor for the toxin cluster expression [270]. Interestingly, the toxin cluster promotor P_*orfX1*_ of non-proteolytic BoNT/E and BoNT/F6 strains in which *botR* is also absent, does not share the highly conserved−35 promoter elements of σ [70] alternative sigma factors. Rather, the promoters share the primary sigma factor recognition sequence (σ^A^), inferring the neurotoxin cluster in these strains may be regulated by σ^A^ rather than an alternate σ [70] family sigma factor [270].

The neurotoxin cluster can be found on different genetic elements; chromosomal, bacteriophage or plasmid borne. *C. botulinum* strains producing C and D or C/D, D/C mosaic toxin types are encoded on bacteriophages, which tend not to integrate into the genome but exist as a plasmid prophage. The prophages exhibit unstable lysogeny, known as pseudo-lysogeny, and can be easily lost through passage resulting in a non-toxigenic strain [271,272]. Alternatively, *bont* genes, including those of *C. botulinum* Group I, II, and IV strains, are frequently found on plasmids ranging in size from ~ 48kb for pCLL of Eklund 17B to ~ 270 kb for pCLJ of strain 657 [273–275]. The presence of the toxin gene on a plasmid is widespread with serotypes BoNT A, B, E, F, and G and in strains carrying multiple BoNT types [273,276–279]. The *bontB* gene in particular is found to be often encoded on a plasmid, in one study 32 out of 60 strains BoNT/B producing strains carried the *bontB* as such [275]. It has been demonstrated that these *bont*-bearing plasmids can be transferred via conjugation across the different physiological Groups of *C. botulinum* and even into nontoxigenic clostridial strains [280,281]. A rare integration event has recently been identified in which an entire *bont-*harbouring plasmid was inserted into the chromosome in three distinct species by homologous recombination [282].

When chromosomally encoded, the cluster appears to be located at specific positions in the genome with unifying characteristics [283,284]. Noticeably, regions in the cluster neighborhood frequently contain insertion sequence (IS) elements or genes associated with transposition or recombination. IS elements usually encode transposases, which mediate their mobility. The IS elements flanking Group I neurotoxin clusters are, however, no longer intact suggesting the insertion of the cluster occurred early in the strain’s evolution [273]. The presence of flagellin genes in close proximity to the neurotoxin cluster in some strains is in line with the observation that they appear to be recombination hotspots in other bacterial species [285,286]. Through sequence analysis of multiple *C. botulinum* strains, it has been shown that the BoNT gene clusters and the rest of the respective genomes evolved independently of one another [91,287]. This evidence has compounded the role of horizontal gene transfer in the acquisition and evolution of BoNT producing strains, giving reason for the diversity seen within and between the different groups of bacteria able to produce the BoNT.

The expression and production of BoNT is growth phase dependent, peaking at late exponential-early stationary phase and rapidly declining through stationary phase [288,289]. In 2006, environmental temperature was shown to control BotR regulation, and since then several positive and negative regulatory systems have been described responsible for the tight control of *bont* expression [290–292]. Evidence suggests that the agr quorum sensing system has a regulatory effect on BoNT production, and numerous two component regulatory systems (TCSs) have been found to both positively and negatively regulate toxin expression [293–295]. The production of BoNT is also influenced by nutritional availability, with increased levels of arginine repressing BoNT expression and conversely a decrease in glucose concentration also reducing BoNT expression [296–299]. The approximate 1000-fold reduction in BoNT/A production in the presence of high concentrations of arginine was found to be a due to a pH shift to more alkaline conditions as an indirect consequence of the catabolism of arginine. It was hypothesized that the production of neurotoxin is regulated on a post-translational level by an uncharacterized pH-dependent protease produced by the bacteria that only functions under alkaline conditions [300]. Regulation of metabolism and BoNT expression have been shown to be linked by the global regulator, CodY, which binds to the *ntnh-bontA* promoter region to positively regulate its expression [301]. The regulation of toxin production by nutrient and quorum sensing signals was integrated into a computational model of toxin production by Ihekwaba and colleagues in 2016 [302].

## Sporulation and germination of *C. botulinum*

In addition to the neurotoxin, a key virulence factor in the pathogenesis of *C. botulinum* and prevalence of botulism, is the sporulation and germination survival strategy. This capability enables the bacterium to protect itself from adverse environmental conditions as a dormant and metabolically inert endospore, returning to vegetative cell growth once conditions become favorable, through the process of germination. It is precisely these processes that enable the ubiquitous environmental persistence of *C. botulinum* and its infiltration and neurotoxin production in the specific environmental niches associated with the different forms of botulism [10].

Owing to the genetic and metabolic diversity between *C. botulinum* Groups I-IV, there exists an array of sporulation and germination mechanisms and kinetics within the species *botulinum*.

### Sporulation

A paradigm for bacterial sporulation has been established from an extensive study of the model spore former, *Bacillus subtilis*, aiding in the prediction and elucidation of the sporulation genes and pathways of Gram-positive spore forming bacteria [303,304].

The factors, which initiate the morphological changes required for the process of sporulation remain ambiguous, but are likely to be stimuli representative of hostile microenvironments, such as extremes of temperature, oxygen, ultraviolet radiation, and desiccation; threatening the survival of cells unable to maintain vegetative growth [305]. The cell to cell communication agrBD quorum sensing system may also play a role in the initiation of sporulation, triggered through assessment of cell population density [293].

The sporulation process is initiated when the DNA-binding response regulator Stage 0 sporulation protein, Spo0A, becomes phosphorylated. In *C. botulinum*, the phosphorylation of Spo0A occurs directly by a yet to be determined orphan sensor histidine kinase [306]. This mechanism is thought to have evolved from an ancestral phosphorelay system, lost through convergent and reductive evolution [307]. Phosphorylated Spo0A activates the transcription of stage I and II sporulation genes as well as regulatory sigma factors. These early-stage genes halt binary cell division, inducing the formation of an asymmetric septum and the morphogenesis of the forespore within the mother cell [308]. Spo0A has been demonstrated to be actively expressed in the exponential growth phase in the Group I *C. botulinum* ATCC 3502, and subsequently the expression of the sigma factors essential for sporulation increases in the stationary phase as expression levels of Spo0A decrease [309,310]. As well as having a role in sporulation initiation, Spo0A is also hypothesized as a regulator of toxin production, having been observed to bind directly to the promoter upstream of the *botE* gene in the Group II type E *C. botulinum* Beluga strain [311].

Late-stage sporulation genes are temporally and spatially separated between the mother cell and forespore, with co-ordinated activities between the distinct compartments. These later stages of sporulation involve the sequential addition of multiple surrounding layers and forespore engulfment by the mother cell. The outer layers of the spore; the cortex, outer membrane, and spore coat contribute to the resistance properties of the spore [312,313].

**Figure 8.**
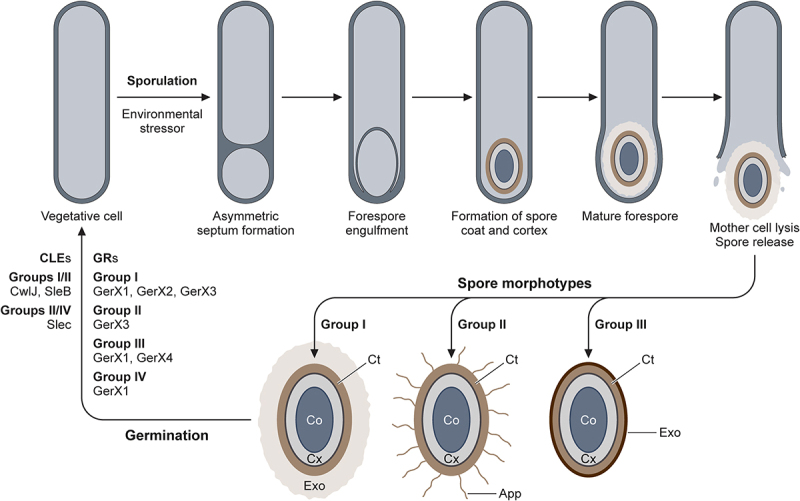
Sporulation and germination of the *C.* *botulinum* Groups. The vegetative cell enters the sporulation program when environmental stressors are encountered. Following lysis of the mother cell, the mature spore is released into the extracellular environment, with diverse spore morphotypes. The elucidated spore morphotypes are presented for isolates of Groups I-III. All spore sub-types possess the fundamental spore core (Co), cortex (Cx) and coat (Ct). Exosporium (Exo) present in Group I (thick and loose fitting) and Group III (thin and tight fitting). Appendages (App) present in Group II spore isolates. The diverse germination mechanisms of Groups I-IV: germinant receptors (GR) and core lytic enzymes (CLE).

Finally, prior to the release of the mature spore, the exosporium envelopes the spore, a structure, which is thought to enhance resilience and pathogenicity through hydrophobic interactions facilitating adhesion. Spores of *C. botulinum* exhibit varying levels of tolerance to environmental stressors, with Group I strains producing highly heat-resistant spores, Group III spores exhibiting moderate heat resistance, and Group II spores the least resistant to heat, hypothesized to be a consequence of diverse exosporia morphotypes (Figure 8) [314].

### Germination

Germination is the process of spore recrudescence to neurotoxin producing vegetative cells, crucial to the pathogenesis of *C. botulinum* and botulism [315]. Improper preservation and storage of food enabling the survival and subsequent germination of *C. botulinum* spores is a potentially lethal series of events in food-borne botulism, where the neurotoxin is ingested. Toxicoinfection may result from the germination of *C. botulinum* spores in environmental favorable conditions, such as the anaerobic environments of a deep wound, or in the underdeveloped infant gut following ingestion of spores, as well as in adults with a depleted gut microbiota.

The germination of spores is triggered by the interaction of small nutrient molecules, termed germinants, with receptors embedded in the inner membrane of the spore [316]. The spores of Groups I and II initiate germination in response to the stereospecific interaction of certain amino acids with germinant receptors [317,318]. Following the binding of a germinant to its cognate germinant receptor, monovalent cations, and dipicolinic acid are released, prior to hydrolysis of the cortex peptidoglycan by core lytic enzymes. This results in core rehydration and vegetative cell outgrowth [319].

As with sporulation, the germination mechanisms of the groups making up the *C. botulinum* species are diverse and not fully understood. Two separate pathways have been hypothesized for Groups I and III and Groups II and IV involving subtly distinct germinant receptors and core lytic enzymes (Figure 8) [317]. Furthermore, the functional germinant receptor essential for spore germination of Group II *C. botulinum* has been identified [320].

## Concluding remarks

Although cases of botulism have decreased over the last decade, *C. botulinum* remains a notable pathogen of importance to public health, not least due to the potential bioterrorism risk and the extensive pharmaceutical applications of the BoNT. The relevance of BoNT in the medical and cosmetic fields has stimulated research interest in pathogens. Due to dangers associated with handling the highly toxic neurotoxin and its listing as a select biological agent, research into this pathogen is restricted to only a few authorized laboratories across the world. This has been the main barrier to elucidating a greater understanding of the pathogen. An established set of genetic tools to produce mutants in the bacteria is now available [321–324] which can allow the development of safe, non-toxigenic strains of *C. botulinum,* which may offer the possibility for further work outside of these select facilities in the future [325].

Central to its pathogenicity and virulence is the BoNT, whose pathological mechanism of action is reliant on inhibition of the release of neurotransmitters at nerve terminals. Although the structural architecture is generally well defined for many serotypes, the molecular mechanisms of the neurotoxins are far from fully elucidated, including the molecular dynamics of the neurotoxin within the cell. The function of the neurotoxin accessory proteins, particularly the OrfX proteins, and their role in virulence remains unknown. Some evidence suggests that the toxin may also have action at the CNS, via retrograde transport of the toxin following entry via the presynaptic nerve membrane. Further research is required to fully understand the mechanism behind this, which may lead to targeted CNS-based therapies in the future. A greater understanding at the molecular level could open the possibilities for further structure-based engineering of the toxin, producing recombinant toxin forms with enhanced clinical efficacy, safer production methods of pharmaceutical BoNTs, and targeted cellular delivery due to BoNTs binding specificity [326–330]. Numerous regulators of neurotoxin expression in the bacterium have been identified, but a full picture of the regulatory network underpinning neurotoxin production would remain useful for public safety measures and pharmaceutical synthesis.

Second only to the neurotoxin in *C. botulinum* virulence factors is the bacteria’s ability to sporulate, allowing persistence through challenging and diverse environments. Gaining a greater understanding of the mechanisms responsible for this crucial part of the pathogen’s life cycle could lead to more preventatives and therapies. Specific germination agents, although well defined for many other clostridia, remain largely unknown in the case of *C. botulinum*; exclusion of these germinant molecules from high-risk foods and preservation methods could be a valuable preventative strategy. Further elucidation of germinant mechanisms and receptors of *C. botulinum* spores could also yield novel preventatives. The development of new botulism treatments and diagnostics has remained an active area and will hopefully provide greater safety to populations in the event of a bioterror attack, as well as allow improved ethical surveillance of imminent risks.

The decreasing cost of next-generation sequencing and resultant availability of high-quality genome sequences has highlighted the genetic diversity within the species. The heterogeneity of neurotoxin gene clusters has given clues regarding the horizontal transfer of the neurotoxin locus between distantly related strains. The single-species taxonomy of 1950s has led to a convoluted classification of *C. botulinum* strains, and there is now a compelling argument for the strict implementation of a more rigorous taxonomic system of nomenclature, as the field continues to discover more strains of this important pathogen.

## Data Availability

Data sharing is not applicable to this article as no new data were created or analysed in this study.

## References

[1] Hauschild AHW. *Clostridium botulinum*. In: Doyle M, editor. Foodborne bacterial pathogens. New York: Marcel Dekker Inc; 1989. pp. 111–28.

[2] Hatheway CL. Botulism. In: Barlows A, WJ Hausler Jr., M Ohashi, A Turano, Lennete EH, editors. Laboratory diagnosis of infectious diseases. New York: Springer; 1988. p. 111–133.

[3] Sobel J. Botulism. Clin Infect Dis. 2005;41(8):1167–1173. 16163636 10.1086/444507

[4] Johnson EA. *Clostridium botulinum*. In: Doyle M, Buchanan R, editors. Food Microbiology. Washington DC: ASM Press; 2019. pp. 487–512.

[5] Johnson EA. *Clostridium botulinum* and the Most Poisonous Poison. In: Gurtler J, Doyle M, Kornacki J, editors. Foodborne Pathogens. Cam, Switzerland: Springer; 2017. pp.553–601.

[6] Rossetto O, Pirazzini M, Fabris F, et al. Botulinum Neurotoxins: Mechanism of Action . Handbook of Experimental Pharmacology. 2021;263 :35–47. 32277300 10.1007/164_2020_355

[7] Hauschild AHW. *Clostridium botulinum*. In: editors, Hauschild A, and K Dodds. Ecology and Control in Foods. 1st ed. Boca Raton: CRC Press; 1993.

[8] Minton NP, Clarke DJ. Clostridia. New York: Plenum; 1989.

[9] Hatheway CL. Toxigenic clostridia. Clinical Microbiology Reviews. 1990;3(1):66–98. 2404569 10.1128/cmr.3.1.66 PMC358141

[10] Peck MW. Biology and Genomic Analysis of *Clostridium botulinum*. Adv Microb Physiol. 2009;55:183–320. 19573697 10.1016/S0065-2911(09)05503-9

[11] Shukla HD, Sharma SK. *Clostridium botulinum*: a bug with beauty and weapon. Crit Rev Microbiol. 2005;31(1):11–18. 15839401 10.1080/10408410590912952

[12] Nigam P, Nigam A. Botulinum toxin. Indian J Dermatol. 2010;55(1):8–14. 20418969 10.4103/0019-5154.60343 PMC2856357

[13] Dorizas A, Krueger N, Sadick NS. Aesthetic uses of the botulinum toxin. Dermatol Clin. 2014;32(1):23–36. 24267419 10.1016/j.det.2013.09.009

[14] Chen S. Clinical uses of botulinum neurotoxins: current indications, limitations and future developments. Toxins (Basel). 2012;4:913–939. 23162705 10.3390/toxins4100913 PMC3496996

[15] Rossetto O, Pirazzini M, Montecucco C. Botulinum neurotoxins: genetic, structural and mechanistic insights. Nature Rev Microbiol. 2014;12(8):535–549. 24975322 10.1038/nrmicro3295

[16] Lund BM, Peck M. *Clostridium botulinum*. In: Lund BM, Baird-Parker AC, GW Gould, editors. The Microbiological Safety and Quality of Food. Maryland, MD, USA: Aspen; 2000. pp. 1057–1109.

[17] Erbguth FJ. From poison to remedy: the chequered history of botulinum toxin. J Neural Transm. 2008;115(4):559–565. 17458494 10.1007/s00702-007-0728-2

[18] Kerner J Neue beobachtungen über die in württemberg so häufig vorfallenden tödlichen vergiftungen durch den genuss geräucherter würste. Tübingen, Oisander 1820.

[19] Erbguth FJ, Naumann M. Historical aspects of botulinum toxin: justinus Kerner (1786-1862) and the sausage poison. Neurology. 1999;53(8):1850. 10563638 10.1212/wnl.53.8.1850

[20] Erbguth FJ. Historical notes on botulism, *Clostridium botulinum*, botulinum toxin, and the idea of the therapeutic use of the toxin. Mov Disord. 2004;19(S8):S2–6. 15027048 10.1002/mds.20003

[21] van Ermengem E. A New Anaerobic Bacillus and Its Relation to Botulism. Clin Infect Dis. 1979;1(4):701–719. 399378

[22] Winslow CE, Broadhurst J, Buchanan RE, et al. THE families and genera of the bacteria preliminary report of the committee of the society of American bacteriologists on characterization and classification of bacterial types. J Bacteriol. 1917;2(5):505. 16558764 10.1128/jb.2.5.505-566.1917 PMC378727

[23] Burke GS. The Occurrence of *Bacillus botulinus* in Nature. J Bacteriol. 1919;4(5):541. 16558851 10.1128/jb.4.5.541-553.1919 PMC378819

[24] Sommer H, Nealon PJ, Snipe PT. Studies on Botulinum Toxin: 4. Dialysis Experiments. J Infect Dis. 1928;43(2):161–166.

[25] Lamanna C, Eklund HW, McElroy OE. Botulinum Toxin (Type A); Including a Study of Shaking with Chloroform as a Step in the Isolation Procedure. J Bacteriol. 1946;52(1):1. PMC518133 16561137

[26] Burgen ASV, Dickens F, Zatman LJ. The action of botulinum toxin on the neuromuscular junction. Journal of Physiology. 1949;109(1–2):10–24. 10.1113/jphysiol.1949.sp004364 PMC1392572 15394302

[27] Schiavo GG, Benfenati F, Poulain B, et al. Tetanus and botulinum-B neurotoxins block neurotransmitter release by proteolytic cleavage of synaptobrevin. Nature. 1992;359(6398):832–835. 1331807 10.1038/359832a0

[28] Schantz EJ, Johnson EA. Botulinum toxin: the story of its development for the treatment of human disease. Perspect Biol Med. 1997;40(3):317–327. 9167258 10.1353/pbm.1997.0032

[29] Pellett S. Learning from the past: historical aspects of bacterial toxins as pharmaceuticals. Curr Opin Microbiol. 2012;15(3):292–299. 22651975 10.1016/j.mib.2012.05.005

[30] Scott AB. Botulinum toxin injection to correct strabism. Trans Am Ophthalmol Soc. 1979;79:924–927. PMC1312202 7043872

[31] Scott AB. Botulinum Toxin Injection into Extraocular Muscles as an Alternative to Strabismus Surgery. Ophthalmol. 1980;87(10):1044–1049. 10.1016/s0161-6420(80)35127-0 7243198

[32] Schantz EJ, Scott AB. Use of crystalline type a botulinum toxin in medical research. In: Lewis E, editor. Biomedical Aspects of Botulism. San Diego, CA: Academic Press; 1981. pp. 143–150.

[33] Ting PT, Freiman A. The story of *Clostridium botulinum*: from food poisoning to Botox. Clin Med. 2004;4:258–261. 10.7861/clinmedicine.4-3-258 PMC4953590 15244362

[34] Monheit GD, Pickett A. AbobotulinumtoxinA: a 25-year history. Aesthet Surg J. 2017;37(suppl_1):S4–11. 10.1093/asj/sjw284 PMC5434488 28388718

[35] Choudhury S, Baker MR, Chatterjee S, et al. Botulinum toxin: an update on pharmacology and newer products in development. Toxins (Basel). 2021;13(1):58. 33466571 10.3390/toxins13010058 PMC7828686

[36] Frevert J, Ahn KY, Park MY, et al. Comparison of botulinum neurotoxin type a formulations in Asia. 2018;11:327. 10.2147/CCID.S160723 PMC6039073 30013379

[37] Duplantier J, A DK, Bavari C, et al. Searching for therapeutics against botulinum neurotoxins: a true challenge for drug discovery. Curr Top Med Chem. 2016;16(21):2330–2349. 27072693 10.2174/1568026616666160413135630

[38] Hill KK, Smith TJ, Helma CH, et al. Genetic diversity among botulinum neurotoxin-producing clostridial strains. J Bacteriol. 2007;189(3):818–832. 17114256 10.1128/JB.01180-06 PMC1797315

[39] Holdeman LV, Brooks JB. Variation among strains of *Clostridium botulinum* and related clostridia. In: Herzberg M, editor. Proceedings of the 1st US-Japan Conference on Toxic Microorganisms. Washington, DC.: U.S. Government Printing Office; 1970. p. 278–286.

[40] Collins C, East E. Phylogeny and taxonomy of the food-borne pathogen *Clostridium botulinum* and its neurotoxins. J Appl Microbiol. 1998;84:5–17. 15244052 10.1046/j.1365-2672.1997.00313.x

[41] Peck MW, Smith TJ, Anniballi F, et al. Historical perspectives and guidelines for botulinum neurotoxin subtype nomenclature. Toxins (Basel). 2017;9(1):38. 28106761 10.3390/toxins9010038 PMC5308270

[42] Hill KK, Smith TJ. Genetic Diversity Within *Clostridium botulinum* Serotypes, Botulinum Neurotoxin Gene Clusters and Toxin Subtypes. Curr Top Microbiol Immunol. 2012;364:1–20. 10.1007/978-3-642-33570-9_1 23239346

[43] Suen JC, Hatheway CL, Steigerwalt AG, et al. *Clostridium argentinense sp*. nov.: a genetically homogeneous group composed of all strains of *Clostridium botulinum* toxin type G and some nontoxigenic strains previously identified as *Clostridium subterminale* or *Clostridium hastiforme*. Int J Bacteriol. 1988;38(4):375–381.

[44] Zhang S, Masuyer G, Zhang J, et al. Identification and characterization of a novel botulinum neurotoxin. Nat Commun. 2017;8(1):1–10. 28770820 10.1038/ncomms14130 PMC5543303

[45] Dover N, Barash JR, Hill KK, et al. Molecular characterization of a novel botulinum neurotoxin type H gene. J Infect Dis. 2014;209(2):192–202. 24106295 10.1093/infdis/jit450

[46] Kalb SR, Baudys J, Raphael BH, et al. Functional Characterization of Botulinum Neurotoxin Serotype H as a Hybrid of Known Serotypes F and a (BoNT F/A). Anal Chem. 2015;87(7):3911–3917. 25731972 10.1021/ac504716v PMC4522910

[47] Maslanka SE, Lúquez C, Dykes JK, et al. A novel botulinum neurotoxin, previously reported as Serotype H, has a hybrid-like structure with regions of similarity to the structures of serotypes a and F and is neutralized with Serotype a antitoxin. J Infect Dis. 2016;213(3):379–385. 26068781 10.1093/infdis/jiv327 PMC4704661

[48] Leuchs J. Beiträge zur Kenntnis des Toxins und Antitoxins des Bacillus botulinus. Zeitschrift für Hygiene und Infektionskrankheiten. 1910;76(1):55–84.

[49] Smith TJ, Lou J, Geren IN, et al. Sequence variation within botulinum neurotoxin serotypes impacts antibody binding and neutralization. Infect Immun. 2005;73(9):5450–5457. 16113261 10.1128/IAI.73.9.5450-5457.2005 PMC1231122

[50] Rossetto O, Montecucco C. Tables of Toxicity of Botulinum and Tetanus Neurotoxins. Toxins (Basel). 2019;11(12):686. 31771110 10.3390/toxins11120686 PMC6950492

[51] Franciosa G, Floridi F, Maugliani A, et al. Differentiation of the gene clusters encoding botulinum neurotoxin type a complexes in *Clostridium botulinum* type A, Ab, and A(B) strains. Appl Environ Microbiol. 2004;70(12):7192–7199. 15574917 10.1128/AEM.70.12.7192-7199.2004 PMC535171

[52] Moriishi K, Koura M, Fujii N, et al. Molecular cloning of the gene encoding the mosaic neurotoxin, composed of parts of botulinum neurotoxin types C1 and D, and PCR detection of this gene from *Clostridium botulinum* type C organisms. Appl Environ Microbiol. 1996;62(2):662. 8593068 10.1128/aem.62.2.662-667.1996 PMC167833

[53] Moriishi K, Koura M, Abe N, et al. Mosaic structures of neurotoxins produced from *Clostridium botulinum* types C and D organisms 1. Biochimica Et Biophysica Acta (BBA) - Gene Structure and Expression. 1996;1307(2):123–126. 8679691 10.1016/0167-4781(96)00006-1

[54] Nakamura K, Kohda T, Umeda K, et al. Characterization of the D/C mosaic neurotoxin produced by *Clostridium botulinum* associated with bovine botulism in Japan. Vet Microbiol. 2010;140:147–154. 19720474 10.1016/j.vetmic.2009.07.023

[55] Schleberger C, Hochmann H, Barth H, et al. Structure and action of the binary C2 toxin from *Clostridium botulinum*. J Mol Biol. 2006;364:705–715. 17027031 10.1016/j.jmb.2006.09.002

[56] Sakaguchi Y, Suzuki T, Yamamoto Y, et al. Genomics of *Clostridium botulinum* group III strains. Res Microbiol. 2015;166:318–325. 25111022 10.1016/j.resmic.2014.07.016

[57] Zornetta I, Azarnia Tehran D, Arrigoni G, et al. The first non *Clostridial botulinum*-like toxin cleaves VAMP within the juxtamembrane domain. Sci Rep. 2016;6. 27443638 10.1038/srep30257 PMC4957215

[58] Zhang S, Lebreton F, Mansfield MJ, et al. Identification of a Botulinum Neurotoxin-like Toxin in a Commensal Strain of *Enterococcus faecium*. Cell Host & Microbe. 2018;23(2):169–176.e6. 29396040 10.1016/j.chom.2017.12.018 PMC5926203

[59] Brunt J, Carter AT, Stringer SC, et al. Identification of a novel botulinum neurotoxin gene cluster in *Enterococcus*. FEBS Lett. 2018;592(3):310–317. 29323697 10.1002/1873-3468.12969 PMC5838542

[60] Wentz TG, Muruvanda T, Lomonaco S, et al. Closed genome sequence of *Chryseobacterium piperi* strain CTMT/ATCC BAA- 1782, a Gram-negative bacterium with clostridial neurotoxin-like coding sequences. Genome Announc. 2017; 5(48). 10.1128/genomeA.01296-17 PMC5722062 29192076

[61] Eleopra R, Tugnoli V, Rossetto O, et al. Different time courses of recovery after poisoning with botulinum neurotoxin serotypes a and E in humans. Neurosci Lett. 1998;256:135–138. 9855358 10.1016/s0304-3940(98)00775-7

[62] Keller JE. Recovery from botulinum neurotoxin poisoning in vivo. Neuroscience. 2006;139(2):629–637. 16490322 10.1016/j.neuroscience.2005.12.029

[63] Seddon HR. Bulbar Paralysis in Cattle Due to the Action of a Toxicogenic Bacillus, with a Discussion on the Relationship of the Condition to Forage Poisoning (Botulism). J Comp Pathol Ther. 1922;35:147–190.

[64] Skarin H, Håfström T, Westerberg J, et al. *Clostridium botulinum* group III: a group with dual identity shaped by plasmids, phages and mobile elements. BMC Genomics. 2011;12(1):185. 21486474 10.1186/1471-2164-12-185 PMC3098183

[65] Smith T, Williamson CHD, Hill K, et al. Botulinum neurotoxin-producing bacteria. Isn’t it time that we called a species a species? MBio. 2018;9(5). 10.1128/mBio.01469-18 PMC6156192 30254123

[66] Tamura K, Stecher G, Kumar S. MEGA11: molecular Evolutionary Genetics Analysis Version 11. Mol Biol Evol. 2021;38(7):3022–3027. 33892491 10.1093/molbev/msab120 PMC8233496

[67] Janik E, Ceremuga M, Bijak JS, et al. Biological toxins as the potential tools for bioterrorism. Int J Mol Sci. 2019;20. 30857127 10.3390/ijms20051181 PMC6429496

[68] Carus WS. Bioterrorism and Biocrimes: the Illicit Use of Biological Agents Since 1900. . Washington DC, USA: Center for Counterproliferation Research, National Defense University; 2001. p. 209.

[69] U.K. Government. Schedule 5 Pathogens and toxins [Internet]. 2001 [cited 20 October 2022]. Available from: http://www.legislation.gov.uk/ukpga/2001/24/contents.

[70] Centers for Disease Control and Prevention (CDC). Bioterrorism Agents/Diseases [Internet]. 2018 [cited 20 October 2022]; Available from: https://emergency.cdc.gov/agent/agentlist-category.asp.

[71] Josko D. Botulin toxin: a weapon in terrorism. Clin. Lab Sci. 2004;17:30–34. 15011978

[72] Patocka J, Splino M, Merka V. Botulism and bioterrorism: how serious is this problem? Acta medica (Hradec Králové). Acta Medica (Hradec Kralove). 2005;48(1):23–28. 16080379

[73] Arnon SS, Schechter R, V IT, et al. Botulinum toxin as a biological weapon. JAMA. 2001;285:1059–1070. 11209178 10.1001/jama.285.8.1059

[74] Smith TJ, Roxas-Duncan V, Smith L. Botulinum neurotoxins as biothreat agents. J Bioterror Biodef. 2012;2:S2:003.

[75] Zilinskas RA. Iraq’s Biological Weapons. JAMA. 1997;278:418–424. 9244334

[76] Villar RG, Elliott SP, Davenport KM. Botulism: the Many Faces of Botulinum Toxin and its Potential for Bioterrorism. Infect Dis Clin North Am. 2006;20:313–327. 16762741 10.1016/j.idc.2006.02.003

[77] Kazdobina IS. Stability of botulin toxins in solutions and beverages. Gig Sanit. 1995;1:9–12. 7744287

[78] Siegel LS. Destruction of Botulinum Toxins in Food and Water. In: Hauschild A, Dodds K, editors. Ecology and Control in Foods. 1st ed. Boca Raton: CRC Press; 1993. pp. 323–341.

[79] Cenciarelli O, Riley PW, Baka A. Biosecurity Threat Posed by Botulinum Toxin. Toxins (Basel). 2019;11(12):681. 31757074 10.3390/toxins11120681 PMC6950065

[80] Johnson EA, Montecucco C. Botulism. In: Andrew GE, editor. Handbook of Clinical Neurology. Vol. 91. Amsterdam, NL: Elsevier; 2008. p. 333–368. DOI:10.1016/S0072-9752(07)01511-4 18631849

[81] Centers for Disease Control and Prevention (CDC). National Botulism Surveillance Summary, 2017 [Internet]. [cited 20 Occtober 2022]. Available from: https://www.cdc.gov/botulism/surv/2017/index.html

[82] Gangarosa EJ, Donadio JA, Armstrong RW, et al. Botulism in the United States, 1899–1969. Am J Epidemiol. 1971;93(2):93–101. 4925448 10.1093/oxfordjournals.aje.a121239

[83] Shapiro RL. Botulism in the United States: a clinical and epidemiologic review. Ann internal med. 1998;129(3):221–228. 9696731 10.7326/0003-4819-129-3-199808010-00011

[84] Sobel J, Tucker N, Sulka A, et al. Foodborne Botulism in the United States, 1990–2000. Emerg Infect Dis. 2004;10(9):1606–1611. 15498163 10.3201/eid1009.030745 PMC3320287

[85] Critchley EM. A comparison of human and animal botulism: a review. J R Soc Med. 1991;84(5):295. 2041009 10.1177/014107689108400516 PMC1293230

[86] Hughes JM, Blumenthal JR, Merson MH, et al. Clinical features of types a and B food-borne botulism. Ann Intern Med. 1981;95:442–445. 7283294 10.7326/0003-4819-95-4-442

[87] Lund BM, Peck MW. Heat resistance and recovery of spores of non-proteolytic *Clostridium botulinum* in relation to refrigerated, processed foods with an extended shelf-life. J Appl Bacteriol. 1994;76:115S–128S. 10.1111/j.1365-2672.1994.tb04363.x 8047905

[88] Peck MW. *Clostridium botulinum* and the safety of refrigerated processed foods of extended durability. Trends Food Sci Technol. 1997;8:186–192.

[89] McLauchlin J, Grant KA, Little CL. Food-borne botulism in the United Kingdom. J Public Health. 2006;28(4):337–342. 10.1093/pubmed/fdl053 16917124

[90] International Commission on Microbiological Specifications for Foods (ICMSF). *Clostridium botulinum*. In: Roberts TA, Baird-Parker AC, Tompkin RB, editors. Microorganisms in Foods 5: characteristics of microbial pathogens. London, UK: Blackie Academic & Professional; 1996. p. 68–111.

[91] Carter AT, Peck MW. Genomes, neurotoxins and biology of *Clostridium botulinum* Group I and Group II. Res Microbiol. 2015;166(4):303–317. 25445012 10.1016/j.resmic.2014.10.010 PMC4430135

[92] Peck MW. *Clostridium botulinum* and the safety of minimally heated, chilled foods: an emerging issue? J Appl Microbiol. 2006;101(3):556–570. 16907806 10.1111/j.1365-2672.2006.02987.x

[93] Peck MW, Stringer SC. The safety of pasteurised in-pack chilled meat products with respect to the foodborne botulism hazard. Meat Sci. 2005;70(3):461–475. 22063745 10.1016/j.meatsci.2004.07.019

[94] Stumbo CR, Purohit KS, Ramakrishnan TV. Thermal process lethality guide for low‐acid foods in metal containers. J Food Sci. 1975;40(6):1316–1323.

[95] Lindström M, Kiviniemi K, Korkeala H. Hazard and control of group II (non-proteolytic) *Clostridium botulinum* in modern food processing. Int J Food Microbiol. 2006;108:92–104. 16480785 10.1016/j.ijfoodmicro.2005.11.003

[96] Juliao PC, Maslanka S, Dykes J, et al. National outbreak of type a foodborne botulism associated with a widely distributed commercially canned hot dog chili sauce. Clin Infect Dis. 2013;56(3):376–382. 23097586 10.1093/cid/cis901 PMC4538949

[97] Rocke TE, Samuel MD. Water and Sediment Characteristics Associated with Avian Botulism Outbreaks in Wetlands. J Wildl Manage. 1999;63(4):1249.

[98] Espelund M, Klaveness D. Botulism outbreaks in natural environments – an update. Front Microbiol. 2014;5:287. 24966853 10.3389/fmicb.2014.00287 PMC4052663

[99] Duncan RM, Jensen WI. A relationship between avian carcasses and living invertebrates in the epizootiology of avian botulism. J Wildl Dis. 1976;12:116–126. 1255907 10.7589/0090-3558-12.1.116

[100] Johnson AL, McAdams-Gallagher SC, Aceto H. Accuracy of a Mouse Bioassay for the Diagnosis of Botulism in Horses. J Vet Intern Med. 2016;30:1293–1299. 27108763 10.1111/jvim.13950 PMC5074318

[101] Cagan E, Peker E, Dogan M, et al. Infant Botulism. Eurasian J Med. 2010;42(2):92. 25610131 10.5152/eajm.2010.25 PMC4261338

[102] Fox CK, Keet CA, Strober JB. Recent advances in infant botulism. Pediatr Neurol. 2005;32(3):149–154. 15730893 10.1016/j.pediatrneurol.2004.10.001

[103] Tanzi MG, Gabay MP. Association between honey consumption and infant botulism. Pharmacotherapy. 2002;22(11):1479–1483. 12432974 10.1592/phco.22.16.1479.33696

[104] Arnon SS, Midura TF, Damus K, et al. Honey and other environmental risk factors for infant botulism. J Paediatr. 1979;94(2):331–336. 10.1016/s0022-3476(79)80863-x 368301

[105] Aureli P, Franciosa G, Fenicia L. Infant botulism and honey in Europe: a commentary. Pediatr Infect Dis J. 2002;21(9):866–868. 12352811 10.1097/00006454-200209000-00016

[106] Brett MM, McLauchlin J, Harris A, et al. A case of infant botulism with a possible link to infant formula milk powder: evidence for the presence of more than one strain of *Clostridium botulinum* in clinical specimens and food. J Med Microbiol. 2005;54:769–776. 16014431 10.1099/jmm.0.46000-0

[107] Nevas M, Lindstrom M, Virtanen A, et al. Infant botulism acquired from household dust presenting as sudden infant death syndrome. J Clin Microbiol. 2005;43(1):511–513. 15635031 10.1128/JCM.43.1.511-513.2005 PMC540168

[108] Midura TF, Arnon SS. Infant botulism. Identification of *Clostridium botulinum* and its toxins in faeces. Lancet. 1976;308(7992):934–936. 10.1016/s0140-6736(76)90894-1 62164

[109] Koepke R, Sobel J, Arnon SS. Global occurrence of infant botulism, 1976-2006. Pediatrics. 2008;122(1):122. 10.1542/peds.2007-1827 18595978

[110] Centers for Disease Control and Prevention (CDC). National Botulism Surveillance [Internet]. 2018 [cited 20th October 2022]. Available from: https://www.cdc.gov/botulism/surveillance.html.

[111] Davis JB, Mattman LH, Wiley M. *Clostridium botulinum* in a Fatal Wound Infection. J Am Med Assoc. 1951;146:646–648. 14832034 10.1001/jama.1951.63670070006009d

[112] Merson MH, Dowell VR. Epidemiologic, Clinical and Laboratory Aspects of Wound Botulism. N Engl J Med. 1973;289:1005–1010. 4582478 10.1056/NEJM197311082891904

[113] Elston HR, Wang M, Loo LK. Arm abscesses caused by *Clostridium botulinum*. J Clin Microbiol. 1991;29:2678. 1774288 10.1128/jcm.29.11.2678-2679.1991 PMC270406

[114] Brett MM, Hood J, Brazier JS, et al. Soft tissue infections caused by spore-forming bacteria in injecting drug users in the United Kingdom. Epidemiol Infect. 2005;133:575–582. 16050501 10.1017/s0950268805003845 PMC2870283

[115] Kuehn B. Wound Botulism Outbreak. JAMA. 2019;321(6):538. 10.1001/jama.2019.0050 30747972

[116] Werner SB, Passaro D, McGee J, et al. Wound Botulism in California, 1951–1998: recent Epidemic in Heroin Injectors. Clin Infect Dis. 2000;31:1018–1024. 11049786 10.1086/318134

[117] Passaro DJ, Werner SB, McGee J, et al. Wound botulism associated with black tar heroin among injecting drug users. J Am Med Assoc. 1998;279:859–863. 10.1001/jama.279.11.859 9516001

[118] McCroskey LM, Hatheway CL. Laboratory findings in four cases of adult botulism suggest colonization of the intestinal tract. J Clin Microbiol. 1988;26(5):1052. 3290234 10.1128/jcm.26.5.1052-1054.1988 PMC266519

[119] Fenicia L, Anniballi F, Aureli P. Intestinal toxemia botulism in Italy, 1984-2005. Eur J Clin Microbiol Infect Dis. 2007;26:385–394. 17516104 10.1007/s10096-007-0301-9

[120] Harris RA, Anniballi F, Austin JW. Adult Intestinal Toxemia Botulism. Toxins (Basel). 2020;12(2):81./81. 10.3390/toxins12020081 PMC7076759 31991691

[121] Bakheit AMO, Ward CD, McLellan DL. Generalised botulism-like syndrome after intramuscular injections of botulinum toxin type A: a report of two cases [3]. J Neurol Neurosurg Psychiatry. 1997;62(2):198. 10.1136/jnnp.62.2.198 PMC486736 9048725

[122] Chertow DS, Tan ET, Maslanka SE, et al. Botulism in 4 adults following cosmetic injections with an unlicensed, highly concentrated botulinum preparation. JAMA. 2006;296(20):2476–2479. 17119144 10.1001/jama.296.20.2476

[123] Crowner BE, Brunstrom JE, Racette BA. Iatrogenic botulism due to therapeutic botulinum toxin a injection in a pediatric patient. Clin Neuropharmacol. 2007;30(5):310–313. 17909312 10.1097/WNF.0b013e31804b1a0d

[124] Holzer E. Botulism caused by inhalation. Medizinische Klin. 1962;57:1735–1738. 13961505

[125] Park JB, Simpson LL. Inhalational poisoning by botulinum toxin and inhalation vaccination with its heavy-chain component. Infect Immun. 2003;71(3):1147–1154. 12595426 10.1128/IAI.71.3.1147-1154.2003 PMC148837

[126] Pitt MLM, LeClaire RD. Pathogenesis by Aerosol. In: Lebeda F , Korch G, Lindler L, editors. Biological Weapons Defense. Totowa, NJ: Humana Press; 2005. pp. 65–78.

[127] O’Horo JC, Harper EP, El Rafei A, et al. Efficacy of Antitoxin Therapy in Treating Patients with Foodborne Botulism: a Systematic Review and Meta-analysis of Cases, 1923-2016. Clin Infect Dis. 2017;66:S43–56. 29293927 10.1093/cid/cix815 PMC5850555

[128] Rao AK, Sobel J, Chatham-Stephens K, et al. Clinical Guidelines for Diagnosis and Treatment of Botulism, 2021. MMWR Recommendations Rep. 2021;70(2):1. 10.15585/mmwr.rr7002a1 PMC8112830 33956777

[129] Yu PA, Lin NH, Mahon BE, et al. Safety and Improved Clinical Outcomes in Patients Treated with New Equine-Derived Heptavalent Botulinum Antitoxin. Clin Infect Dis. 2018;66(suppl_1):S57–64. 10.1093/cid/cix816 PMC5866099 29293928

[130] Barker D, Gillum KT, Niemuth NA, et al. Therapeutic efficacy of equine botulism heptavalent antitoxin against all seven botulinum neurotoxins in symptomatic guinea pigs. PLoS ONE. 2019;14:e0222670. DOI:10.1371/journal.pone.0222670 PMC6748678 31527885

[131] Lonati D, Schicchi A, Crevani M, et al. Foodborne Botulism: clinical diagnosis and medical treatment. Toxins (Basel). 2020;12(8):509. 32784744 10.3390/toxins12080509 PMC7472133

[132] Ben David A, Barnea A, Torgeman A, et al. Immunologic and Protective Properties of Subunit- vs. Whole Toxoid-Derived Anti-Botulinum Equine Antitoxin. Vaccines. 2022;10(9):1522. 36146601 10.3390/vaccines10091522 PMC9506527

[133] US Food and Drug Administration (FDA). Approval History, Letters, Reviews, and Related Documents - BAT (Botulism Antitoxin Heptavalent (A B, C D, E F, G)) (Equine) [Internet]. 2013 [cited 20th October 2022]; Available from: https://www.fda.gov/vaccines-blood-biologics/approved-blood-products/bat-botulism-antitoxin-heptavalent-b-c-d-e-f-g-equine

[134] Atassi MZ. Immune recognition of BoNTs a and B: how anti-toxin antibodies that bind to the heavy chain obstruct toxin action. Toxicon. 2009;54(5):600–613. 19285100 10.1016/j.toxicon.2009.02.034

[135] Tacket CO, Shandera WX, Mann JM, et al. Equine antitoxin use and other factors that predict outcome in type a foodborne botulism. Am j med. 1984;76(5):794–798. 6720725 10.1016/0002-9343(84)90988-4

[136] Black RE, Gunn RA. Hypersensitivity reactions associated with botulinal antitoxin. Am j med. 1980;69(4):567–570. 7191633 10.1016/0002-9343(80)90469-6

[137] Pirazzini M, Rossetto O. Challenges in searching for therapeutics against Botulinum Neurotoxins. Expert Opin Drug Discov. 2017;12(5):497–510. 28271909 10.1080/17460441.2017.1303476

[138] Schussler E, Sobel J, Hsu J, et al. Workgroup Report by the Joint Task Force Involving American Academy of Allergy, Asthma & Immunology (AAAAI); Food Allergy, Anaphylaxis, Dermatology and Drug Allergy (FADDA) (Adverse Reactions to Foods Committee and Adverse Reactions to Drugs, Biologicals, and Latex Committee); and the Centers for Disease Control and Prevention Botulism Clinical Treatment Guidelines Workgroup—Allergic Reactions to Botulinum Antitoxin: a Systematic Review. Clin Infect Dis an off Publ Infect Dis Soc Am. 2018;66:S65. 10.1093/cid/cix827 PMC5850017 29293931

[139] Arnon SS, Schechter R, Maslanka SE, et al. Human Botulism Immune Globulin for the Treatment of Infant Botulism. N Engl J Med. 2006;354(5):462–471. 16452558 10.1056/NEJMoa051926

[140] US Food and Drug Administration (FDA). BabyBIG [Internet]. 2003 [cited 20th October 2022]; Available from: https://www.fda.gov/vaccines-blood-biologics/approved-blood-products/babybig

[141] Fenicia L, Anniballi F. Infant botulism. Ann Ist Super Sanita. Annali dell’Istituto superiore di sanita. 2009;45(2):134–146. 19636165

[142] Payne JR, Khouri JM, Jewell NP, et al. Efficacy of Human Botulism Immune Globulin for the Treatment of Infant Botulism: the First 12 Years Post Licensure. J Paediatr. 2018;193:172–177. 10.1016/j.jpeds.2017.10.035 29229452

[143] Schulte M, Hamsen U, Schildhauer TA, et al. Effective and rapid treatment of wound botulism, a case report. BMC Surg. 2017;17(1):103. 29073888 10.1186/s12893-017-0300-4 PMC5658925

[144] Brook I. Infant botulism. Journal of Perinatology. 2007;27(3):175–180. 17314986 10.1038/sj.jp.7211651

[145] Arnon SS. Botulism as an intestinal toxemia. In: S BM, RJ PD GH GR, editors. Infections of the gastrointestinal tract. New York: Raven Press; 1995. pp. 257–271.

[146] Fagan RP, Neil KP, Sasich R, et al. Initial Recovery and Rebound of Type F Intestinal Colonization Botulism After Administration of Investigational Heptavalent Botulinum Antitoxin. Clin Infect Dis. 2011;53(9):e125–8. 21896700 10.1093/cid/cir550

[147] Rasetti-Escargueil C, Popoff MR. Antibodies and vaccines against botulinum toxins: available measures and novel approaches. Toxins (Basel). 2019;11:528. DOI:10.3390/toxins11090528 31547338 PMC6783819

[148] Thanongsaksrikul J, Chaicumpa W. Botulinum neurotoxins and botulism: a novel therapeutic approach. Toxins (Basel). 2011;3(5):469–488. 22069720 10.3390/toxins3050469 PMC3202833

[149] Nowakowski A, Wang C, Powers DB, et al. Potent neutralization of botulinum neurotoxin by recombinant oligoclonal antibody. Proc Natl Acad Sci, USA. 2002;99(17):11346–11350. 12177434 10.1073/pnas.172229899 PMC123259

[150] Fan Y, Garcia-Rodriguez C, Lou J, et al. A three monoclonal antibody combination potently neutralizes multiple botulinum neurotoxin serotype F subtypes. PLoS ONE. 2017;12:e0174187. DOI:10.1371/journal.pone.0174187 28323873 PMC5360321

[151] Snow DM, Riling K, Kimbler A, et al. Safety and Pharmacokinetics of a Four Monoclonal Antibody Combination Against Botulinum C and D Neurotoxins. Antimicrob Agents Chemother. 2019;63:e01270–19. DOI:10.1128/AAC.01270-19 31591130 PMC6879217

[152] Matsumura T, Amatsu S, Misaki R, et al. Fully Human Monoclonal Antibodies Effectively Neutralizing Botulinum Neurotoxin Serotype B. Toxins (Basel). 2020;12(5):12. 10.3390/toxins12050302 PMC7291131 32392791

[153] Garcia-Rodriguez C, Razai A, Geren IN, et al. A Three Monoclonal Antibody Combination Potently Neutralizes Multiple Botulinum Neurotoxin Serotype E Subtypes. Toxins (Basel). 2018;10(3):10. 10.3390/toxins10030105 PMC5869393 29494481

[154] Godakova SA, Noskov AN, Vinogradova ID, et al. Camelid VHHs Fused to Human Fc Fragments Provide Long Term Protection Against Botulinum Neurotoxin a in Mice. Toxins (Basel). 2019;11:464. DOI:10.3390/toxins11080464 31394847 PMC6723419

[155] Mukherjee J, Ondeck CA, Tremblay JM, et al. Intramuscular delivery of formulated RNA encoding six linked nanobodies is highly protective for exposures to three Botulinum neurotoxin serotypes. Sci Rep. 2022;12(1):12. 35803998 10.1038/s41598-022-15876-2 PMC9266081

[156] Derkaev AA, Ryabova EI, Esmagambetov IB, et al. rAAV expressing recombinant neutralizing antibody for the botulinum neurotoxin type a prophylaxis. Front Microbiol. 2022;13. 10.3389/fmicb.2022.960937 PMC9551282 36238585

[157] Rusnak JM, Smith LA. Botulinum neurotoxin vaccines: past history and recent developments. Hum Vaccines. 2009;5(12):794–805. 10.4161/hv.9420 19684478

[158] Smith LA. Botulism and vaccines for its prevention. Vaccine. 2009;27 Suppl 4:D33–9. DOI:10.1016/j.vaccine.2009.08.059 19837283

[159] Graham R, Thorp F. The Effect of Formalin on Botulinum Toxins A, B and C. J Immunol. 1929;16(4):391–401.

[160] Keller JE. Characterization of new formalin-detoxified botulinum neurotoxin toxoids. Clin Vaccine Immunol. 2008;15(9):1374–1379. 18667637 10.1128/CVI.00117-08 PMC2546675

[161] Centers for Disease Control and Prevention (CDC). Notice of CDC’s discontinuation of investigational pentavalent (ABCDE) botulinum toxoid vaccine for workers at risk for occupational exposure to botulinum toxins. Morb Mortal Wkly Rep. 2011;60:1454–1455. 22031218

[162] Sundeen G, Barbieri JT. Vaccines against botulism. Toxins (Basel). 2017;9(9):268. 28869493 10.3390/toxins9090268 PMC5618201

[163] Hatami F, Shokouhi S, Mardani M, et al. Early recovery of botulism: one decade of experience. Clin Toxicol. 2021;59(7):628–632. 10.1080/15563650.2020.1844225 33156710

[164] De Paiva A, Meunier FA, Molgó J, et al. Functional repair of motor endplates after botulinum neurotoxin type a poisoning: biphasic switch of synaptic activity between nerve sprouts and their parent terminals. Proc Nat Acad Sci. 1999;96(6):3200–3205. 10077661 10.1073/pnas.96.6.3200 PMC15919

[165] Holland RL, Brown MC. Nerve growth in botulinum toxin poisoned muscles. Neuroscience. 1981;6(6):1167–1179. 7279219 10.1016/0306-4522(81)90081-6

[166] Adler M, Franz DR. Toxicity of botulinum neurotoxin by inhalation: implications in bioterrorism. In: Salem H, Katz S, editors. Aerobiology: the Toxicology of Airborne Pathogens and Toxins. Cambridge, U.K: Royal Society of Chemistry; 2016. pp. 167–185.

[167] Meunier FA, Schiavo G, Molgó J. Botulinum neurotoxins: from paralysis to recovery of functional neuromuscular transmission. J Physiol Paris. 2002;96(1–2):105–113. 11755789 10.1016/s0928-4257(01)00086-9

[168] Tsai YC, Maditz R, Kuo C-L, et al. Targeting botulinum neurotoxin persistence by the ubiquitin-proteasome system. Proc Nat Acad Sci. 2010;107(38):16554–16559. 20823219 10.1073/pnas.1008302107 PMC2944746

[169] Tsai YC, Kotiy A, Kiris E, et al. Deubiquitinating enzyme VCIP135 dictates the duration of botulinum neurotoxin type a intoxication. Proc Nat Acad Sci. 2017;114(26):E5158–66. 28584101 10.1073/pnas.1621076114 PMC5495235

[170] Sen E, Kota KP, Panchal RG, et al. Screening of a Focused Ubiquitin-Proteasome Pathway Inhibitor Library Identifies Small Molecules as Novel Modulators of Botulinum Neurotoxin Type a Toxicity. Front Pharmacol. 2021;12:2659. 10.3389/fphar.2021.763950 PMC8503599 34646144

[171] Rao AK, Lin NH, Jackson KA, et al. Clinical Characteristics and Ancillary Test Results Among Patients with Botulism—United States, 2002–2015. Clin Infect Dis. 2017;66(suppl_1):S4–10. 29293936 10.1093/cid/cix935

[172] Chatham-Stephens K, Fleck-Derderian S, Johnson SD, et al. Clinical Features of Foodborne and Wound Botulism: a Systematic Review of the Literature, 1932-2015. Clin Infect Dis. 2017;66:S11–6. 29293923 10.1093/cid/cix811

[173] Louis ME S, Peck SHS, Bowering D, et al. Botulism from chopped garlic: delayed recognition of a major outbreak. Ann Intern Med. 1988;108:363–368. 3341673 10.7326/0003-4819-108-3-363

[174] Maslanka SE, Solomon HM, Sharma S, et al. *Clostridium botulinum* and its toxins. In: Tortorello M, F Downes, S Doores, K Ito, and Y Salfinger, editors. Compendium of methods for the microbiological examination of foods . Washington, DC: American Public Health Association; 2013. p. Chapter 32:1–11. DOI:10.2105/MBEF.0222.037

[175] Solomon HM, Lilly TJ. Chapter 17: *Clostridium Botulinum*. In: Jackson GJ , Merker RI, Bandler R, editors. Bacteriological Analytical Manual. 8th Ed. College Park, MD, USA: U.S. Food and Drug Administration (FDA), Centre for Food Safety and Applied Nutrition; 2001. https://www.fda.gov/food/laboratory-methods-food/bam-chapter-17-clostridium-botulinum

[176] Lindström M, Korkeala H. Laboratory diagnostics of botulism. Clin. Clinical Microbiology Reviews. 2006;19(2):298–314. 16614251 10.1128/CMR.19.2.298-314.2006 PMC1471988

[177] Taylor K. Botulinum toxin testing on animals is still a Europe-wide issue. ALTEX. 2019;36(1):81–90. 30303513 10.14573/altex.1807101

[178] Kakinuma H, Maruyama H, Yamakawa K, et al. Application of nested polymerase chain reaction for the rapid diagnosis of infant botulism type B. Pediatr Int. 1997;39(3):346–348. 10.1111/j.1442-200x.1997.tb03750.x 9241898

[179] De Medici D, Anniballi F, Wyatt GM, et al. Multiplex PCR for Detection of Botulinum Neurotoxin-Producing Clostridia in Clinical, Food, and Environmental Samples. Appl Environ Microbiol. 2009;75(20):6457. 19684163 10.1128/AEM.00805-09 PMC2765140

[180] Szabo EA, Pemberton JM, Gibson AM, et al. Application of PCR to a clinical and environmental investigation of a case of equine botulism. J Clin Microbiol. 1994;32(8):1986–1991. 7989554 10.1128/jcm.32.8.1986-1991.1994 PMC263915

[181] Chellapandi P, Prisilla A. PCR-based molecular diagnosis of botulism (types C and D) outbreaks in aquatic birds. Ann Microbiol. 2018;68(12):835–849.

[182] Cai S, Singh BR, Sharma S. Botulism diagnostics: from clinical symptoms to in vitro assays. Crit Rev Microbiol. 2007;33(2):109–125. 17558660 10.1080/10408410701364562

[183] Thirunavukkarasu N, Johnson E, Pillai S, et al. Botulinum Neurotoxin Detection Methods for Public Health Response and Surveillance. Front Bioeng Biotechnol. 2018;6:80. 29988463 10.3389/fbioe.2018.00080 PMC6024544

[184] Wictome M, Newton KA, Jameson K, et al. Development of in vitro assays for the detection of botulinum toxins in foods. FEMS Immunology & Medical Microbiology. 1999;24(3):319–323. 10397317 10.1111/j.1574-695X.1999.tb01300.x

[185] Cheng LW, Land KM, Stanker LH. Chapter 1: Presence of Botulinum Neurotoxins in Food and Other Biological Samples. In: Morse SA, editor. Bioterrorism. Online Edition. London, UK: InTechOpen; 2012. p. 1–16. DOI:10.5772/33638

[186] Singh AK, Stanker LH, Sharma SK. Botulinum neurotoxin: where are we with detection technologies? Crit Rev Microbiol. 2013;39(1):43–56. 22676403 10.3109/1040841X.2012.691457

[187] Čapek P, Dickerson TJ. Sensing the deadliest toxin: technologies for botulinum neurotoxin detection. Toxins (Basel). 2010;2(1):24–53. 22069545 10.3390/toxins2010024 PMC3206617

[188] Fernández-Salas E, Wang J, Molina Y, et al. Botulinum Neurotoxin Serotype a Specific Cell-Based Potency Assay to Replace the Mouse Bioassay. PLoS ONE. 2012;7(11):e49516. 23185348 10.1371/journal.pone.0049516 PMC3504020

[189] Pharma M. Press Release: alternative Test Method for Botulinum Neurotoxin Now Approved in Europe [Internet]. 2015 [cited 20th October 2022]; Available from: https://www.merz.com/blog/news/alternative-test-method-for-botulinum-neurotoxin-now-approved-in-europe/

[190] Ipsen Inc. Press Release: ipsen’s Cell-Based Assay Receives Approvals in the E.U. and Switzerland for its Botulinum Toxin [Internet]. 2018 [cited 20th October 2022]; Available from: https://www.ipsen.com/websites/IPSENCOM-PROD/wp-content/uploads/2018/08/28165732/00-IAW-ONLINE-POSITION-STATEMENT_Ipsens-CBA-implementation-2018-08-27.pdf

[191] Pellett S. Progress in Cell Based Assays for Botulinum Neurotoxin Detection. Curr Top Microbiol Immunol. 2013;364:257–285. 23239357 10.1007/978-3-642-33570-9_12 PMC3644986

[192] Koh C-Y, Schaff UY, Piccini ME, et al. Centrifugal microfluidic platform for ultrasensitive detection of botulinum toxin. Anal Chem. 2015;87(2):922–928. 25521812 10.1021/ac504054u PMC4303339

[193] Halliwell J, Gwenin C. A label free colorimetric assay for the detection of active botulinum neurotoxin type a by SNAP-25 conjugated colloidal gold. Toxins (Basel). 2013;5(8):1381–1391. 23925142 10.3390/toxins5081381 PMC3760041

[194] Patel K, Halevi S, Melman P, et al. A novel surface plasmon resonance biosensor for the rapid detection of botulinum neurotoxins. Biosens (Basel). 2017;7(4):32. 10.3390/bios7030032 PMC5618038 28783115

[195] Savage AC, Buckley N, Halliwell J, et al. Botulinum neurotoxin serotypes detected by electrochemical impedance spectroscopy. Toxins (Basel). 2015;7(5):1544–1555. 25954998 10.3390/toxins7051544 PMC4448162

[196] Hobbs RJ, Thomas CA, Halliwell J, et al. Rapid Detection of Botulinum Neurotoxins—A Review. Toxins (Basel). 2019;11(7):11. 31319550 10.3390/toxins11070418 PMC6669533

[197] Smith TJ, Hill KK, Raphael BH. Historical and current perspectives on *Clostridium botulinum* diversity. Res Microbiol. 2015;166(4):290–302. 25312020 10.1016/j.resmic.2014.09.007 PMC11302483

[198] Lacy DB, Tepp W, Cohen AC, et al. Crystal structure of botulinum neurotoxin type a and implications for toxicity. Nat Struct Biol. 1998;5(10):898–902. 9783750 10.1038/2338

[199] Swaminathan S, Eswaramoorthy S. Structural analysis of the catalytic and binding sites of *Clostridium botulinum* neurotoxin B. Nat Struct Biol. 2000;7(8):693–699. 10932256 10.1038/78005

[200] Kumaran D, Eswaramoorthy S, Furey W, et al. Domain organization in *Clostridium botulinum* neurotoxin type E is unique: its implication in faster translocation. J Mol Biol. 2009;386(1):233–245. 19118561 10.1016/j.jmb.2008.12.027

[201] Dekleva ML, Dasgupta BR. Purification and characterization of a protease from *Clostridium botulinum* type a that nicks single-chain type a botulinum neurotoxin into the di-chain form. J Bacteriol. 1990;172(5):2498–2503. 2185224 10.1128/jb.172.5.2498-2503.1990 PMC208889

[202] Pettersen EF, Goddard TD, Huang CC, et al. UCSF ChimeraX: structure visualization for researchers, educators, and developers. Protein Sci. 2021;30:70–82. 32881101 10.1002/pro.3943 PMC7737788

[203] Bandyopadhyay S, Clark AW, DasGupta BR, et al. Role of the heavy and light chains of botulinum neurotoxin in neuromuscular paralysis. J Biol Chem. 1987;262(6):2660–2663. 3029090

[204] Kriegistein KG, DasGupta BR, Henschen AH. Covalent structure of botulinum neurotoxin type A: location of sulfhydryl groups, and disulfide bridges and identification of C-termini of light and heavy chains. Journal of Protein Chemistry. 1994;13(1):49–57. 8011071 10.1007/BF01891992

[205] Montecucco C, Schiavo G. Structure and Function of Tetanus and Botulinum Neurotoxins. Q Rev Biophys. 1995;28(4):423–472. 8771234 10.1017/s0033583500003292

[206] Pirazzini M, Montecucco C, Rossetto O. Toxicology and pharmacology of botulinum and tetanus neurotoxins: an update. Arch Toxicol. 2022;96(6):1521–1539. 35333944 10.1007/s00204-022-03271-9 PMC9095541

[207] Yao G, Zhang S, Mahrhold S, et al. N-linked glycosylation of SV2 is required for binding and uptake of botulinum neurotoxin a. Nature Structural & Molecular Biology. 2016;23(7):656–662. 10.1038/nsmb.3245 PMC5033645 27294781

[208] Muraro L, Tosatto S, Motterlini L, et al. The N-terminal half of the receptor domain of botulinum neurotoxin a binds to microdomains of the plasma membrane. Biochem Biophys Res Commun. 2009;380(1):76–80. 19161982 10.1016/j.bbrc.2009.01.037

[209] Montecucco C, Zanotti G. Botulinum neurotoxin A1 likes it double sweet. Nature Structural & Molecular Biology. 2016;23:619–621. 10.1038/nsmb.3253 27384187

[210] Rummel A. Two feet on the membrane: uptake of clostridial neurotoxins. Curr Top Microbiol Immunol. 2017;406:1–37. 27921176 10.1007/82_2016_48

[211] Brunger AT, Breidenbach MA, Jin R, et al. Botulinum Neurotoxin Heavy Chain Belt as an Intramolecular Chaperone for the Light Chain. PLOS Pathog. 2007;3:e113. 17907800 10.1371/journal.ppat.0030113 PMC1994969

[212] Galloux M, Vitrac H, Montagner C, et al. Membrane interaction of botulinum neurotoxin a translocation (T) domain: the belt region is a regulatory loop for membrane interaction. J Biol Chem. 2008;283:27668–27676. 18693250 10.1074/jbc.M802557200

[213] Fischer A, Montal M. Molecular dissection of botulinum neurotoxin reveals interdomain chaperone function. Toxicon. 2013;75:101–107. 23396042 10.1016/j.toxicon.2013.01.007 PMC3797153

[214] Gu S, Jin R. Assembly and Function of the Botulinum Neurotoxin Progenitor Complex. Curr Top Microbiol Immunol. 2013;364:21–44. 23239347 10.1007/978-3-642-33570-9_2 PMC3875173

[215] Barrett AJ, Rawlings ND. Types and families of endopeptidases. Biochem Soc Trans. 1991;19:707–715. 1783203 10.1042/bst0190707

[216] Gu S, Rumpel S, Zhou J, et al. Botulinum neurotoxin is shielded by NTNHA in an interlocked complex. Science. 2012;335:977–981. 22363010 10.1126/science.1214270 PMC3545708

[217] Jacobson MJ, Lin G, Raphael B, et al. Analysis of neurotoxin cluster genes in *Clostridium botulinum* strains producing botulinum neurotoxin serotype a subtypes. Appl Environ Microbiol. 2008;74:2778–2786. 18326685 10.1128/AEM.02828-07 PMC2394882

[218] Sebaihia M, Peck MW, Minton NP, et al. Genome sequence of a proteolytic (Group I) *Clostridium botulinum* strain Hall a and comparative analysis of the clostridial genomes. Genome Res. 2007;17(7):1082–1092. 17519437 10.1101/gr.6282807 PMC1899119

[219] Lee K, Gu S, Jin L, et al. Structure of a Bimodular Botulinum Neurotoxin Complex Provides Insights into Its Oral Toxicity. PLOS Pathogens. 2013;9(10):e1003690. 24130488 10.1371/journal.ppat.1003690 PMC3795040

[220] Fujinaga Y, Inoue K, Watanabe S, et al. The haemagglutinin of *Clostridium botulinum* type C progenitor toxin plays an essential role in binding of toxin to the epithelial cells of guinea pig small intestine, leading to the efficient absorption of the toxin. Microbiology. 1997;143(12):3841–3847. 9421908 10.1099/00221287-143-12-3841

[221] Fujinaga Y, Inoue K, Nomura T, et al. Identification and characterization of functional subunits of *Clostridium botulinum* type a progenitor toxin involved in binding to intestinal microvilli and erythrocytes. FEBS Lett. 2000;467(2–3):179–183. 10675534 10.1016/s0014-5793(00)01147-9

[222] Matsumura T, Sugawara Y, Yutani M, et al. Botulinum toxin a complex exploits intestinal M cells to enter the host and exert neurotoxicity. Nat Commun. 2015;6(1):6. 10.1038/ncomms7255 PMC4339894 25687350

[223] Matsumura T, Fujinaga Y. Functional Analysis of Botulinum Hemagglutinin (HA). Methods Mol Biol. 2020;2132:191–200. 32306328 10.1007/978-1-0716-0430-4_20

[224] Jin Y, Takegahara Y, Sugawara Y, et al. Disruption of the epithelial barrier by botulinum haemagglutinin (HA) proteins – differences in cell tropism and the mechanism of action between HA proteins of types a or B, and HA proteins of type C. Microbiology. 2009;155(1):35–45. 19118344 10.1099/mic.0.021246-0

[225] Sugawara Y, Matsumura T, Takegahara Y, et al. Botulinum hemagglutinin disrupts the intercellular epithelial barrier by directly binding E-cadherin. J Cell Bio. 2010;189(4):691–700. 20457762 10.1083/jcb.200910119 PMC2872904

[226] Lee K, Zhong X, Gu S, et al. Molecular basis for disruption of E-cadherin adhesion by botulinum neurotoxin a complex. Science. 2014;344(6190):1405–1410. 24948737 10.1126/science.1253823 PMC4164303

[227] Fujinaga Y, Popoff MR. Translocation and dissemination of botulinum neurotoxin from the intestinal tract. Toxicon. 2018;147:13–18. 29074396 10.1016/j.toxicon.2017.10.020

[228] Gustafsson R, Berntsson RP-A, Martínez-Carranza M, et al. Crystal structures of OrfX2 and P47 from a Botulinum neurotoxin OrfX-type gene cluster. FEBS Lett. 2017;591(22):3781–3792. 29067689 10.1002/1873-3468.12889

[229] Lam K-H, Qi R, Liu S, et al. The hypothetical protein P47 of *Clostridium botulinum* E1 strain Beluga has a structural topology similar to bactericidal/permeability-increasing protein. Toxicon. 2018;147:19–26. 29042313 10.1016/j.toxicon.2017.10.012 PMC5902665

[230] Inoue K, Fujinaga Y, Watanabe T, et al. Molecular composition of *Clostridium botulinum* type a progenitor toxins. Infect Immun. 1996;64(5):1589–1594. 8613365 10.1128/iai.64.5.1589-1594.1996 PMC173966

[231] Simpson L. The life history of a botulinum toxin molecule. Toxicon. 2013;68:40–59. 23518040 10.1016/j.toxicon.2013.02.014

[232] Montecucco C. How do tetanus and botulinum toxins bind to neuronal membranes? Trends Biochem Sci. 1986;11(8):314–317.

[233] Rummel A. Double Receptor Anchorage of Botulinum Neurotoxins Accounts for their Exquisite Neurospecificity. Curr Top Microbiol Immunol. 2013;364:61–90. 23239349 10.1007/978-3-642-33570-9_4

[234] Pirazzini M, Rossetto O, Bolognese P, et al. Double anchorage to the membrane and intact inter-chain disulfide bond are required for the low pH induced entry of tetanus and botulinum neurotoxins into neurons. Cell Microbiol. 2011;13(11):1731–1743. 21790947 10.1111/j.1462-5822.2011.01654.x

[235] Cottone G, Chiodo L, Maragliano L, et al. In Silico Conformational Features of Botulinum Toxins A1 and E1 According to Intraluminal Acidification. Toxins (Basel). 2022;14(9):644. 2022. . 36136581 10.3390/toxins14090644 PMC9500700

[236] Moriyama Y, Futai M. H±atpase, a primary pump for accumulation of neurotransmitters, is a major constituent of brain synaptic vesicles. Biochem Biophys Res Commun. 1990;173:443–448. 1979489 10.1016/s0006-291x(05)81078-2

[237] Fischer A. Synchronized Chaperone Function of Botulinum Neurotoxin Domains Mediates Light Chain Translocation into Neurons. Curr Top Microbiol Immunol. 2013;364:115–137. 23239351 10.1007/978-3-642-33570-9_6

[238] Koriazova LK, Montal M. Translocation of botulinum neurotoxin light chain protease through the heavy chain channel. Nat Struct Biol. 2003;10(1):13–18. 12459720 10.1038/nsb879

[239] Hoch DH, Romero-Mira M, Ehrlich BE, et al. Channels formed by botulinum, tetanus, and diphtheria toxins in planar lipid bilayers: relevance to translocation of proteins across membranes. Proc Nat Acad Sci. 1985;82(6):1692–1696. 3856850 10.1073/pnas.82.6.1692 PMC397338

[240] Fischer A, Montal M. Crucial role of the disulfide bridge between botulinum neurotoxin light and heavy chains in protease translocation across membranes. J Biol Chem. 2007;282:29604–29611. 17666397 10.1074/jbc.M703619200

[241] Pirazzini M, Bordin F, Rossetto O, et al. The thioredoxin reductase-thioredoxin system is involved in the entry of tetanus and botulinum neurotoxins in the cytosol of nerve terminals. FEBS Lett. 2013;587(2):150–155. 23178719 10.1016/j.febslet.2012.11.007

[242] Sutton RB, Fasshauer D, Jahn R, et al. Crystal structure of a SNARE complex involved in synaptic exocytosis at 2.4 Å resolution. Nature. 1998;395(6700):347–353. 9759724 10.1038/26412

[243] Jahn R, Scheller RH. Snares — engines for membrane fusion. Nat Rev Mol Cell Biol. 2006;7(9):631–643. 16912714 10.1038/nrm2002

[244] Südhof TC, Rizo J. Synaptic vesicle exocytosis. Cold Spring Harbor Perspect Biol. 2011;3(12):3. 10.1101/cshperspect.a005637 PMC3225952 22026965

[245] Binz T. Clostridial Neurotoxin Light Chains: devices for SNARE Cleavage Mediated Blockade of Neurotransmission. Curr Top Microbiol Immunol. 2013;364:139–157. 23239352 10.1007/978-3-642-33570-9_7

[246] Rossetto O, Schiavo G, Montecucco C, et al. SNARE motif and neurotoxins. Nature. 1994;372(6505):415–416. 7984234 10.1038/372415a0

[247] Shoemaker CB, Oyler GA. Persistence of Botulinum neurotoxin inactivation of nerve function. Curr Top Microbiol Immunol. 2013;364:179–196. 23239354 10.1007/978-3-642-33570-9_9 PMC3888862

[248] Keller JE, Neale EA. The Role of the Synaptic Protein SNAP-25 in the Potency of Botulinum Neurotoxin Type a. J Biol Chem. 2001;276(16):13476–13482. 11278807 10.1074/jbc.M010992200

[249] Pantano S, Montecucco C. The blockade of the neurotransmitter release apparatus by botulinum neurotoxins. Cell Mol Life Sci. 2014;71(5):793–811. 23749048 10.1007/s00018-013-1380-7 PMC11113401

[250] Pirazzini M, Rossetto O, Eleopra R, et al. Botulinum neurotoxins: biology, pharmacology, and toxicology. Pharmacol Rev. 2017;69:200–235. 28356439 10.1124/pr.116.012658 PMC5394922

[251] Caleo M, Schiavo G. Central effects of tetanus and botulinum neurotoxins. Toxicon. 2009;54(5):593–599. 19264088 10.1016/j.toxicon.2008.12.026

[252] Restani L, Novelli E, Bottari D, et al. Botulinum neurotoxin a impairs neurotransmission following retrograde transynaptic transport. Traffic. 2012;13(8):1083–1089. 22519601 10.1111/j.1600-0854.2012.01369.x

[253] Marchand-Pauvert V, Aymard C, Giboin L-S, et al. Beyond muscular effects: depression of spinal recurrent inhibition after botulinum neurotoxin a. Journal of Physiology. 2013;591(4):1017. 23045348 10.1113/jphysiol.2012.239178 PMC3591712

[254] Aymard C, Giboin L-S, Lackmy-Vallée A, et al. Spinal plasticity in stroke patients after botulinum neurotoxin a injection in ankle plantar flexors. Physiological Reports. 2013;1(6):e00173. 24400171 10.1002/phy2.173 PMC3871484

[255] Mazzocchio R, Caleo M. More than at the neuromuscular synapse: actions of botulinum neurotoxin a in the central nervous system. Neuroscientist. 2015;21(1):44–61. 24576870 10.1177/1073858414524633

[256] Marinelli S, Vacca V, Ricordy R, et al. The Analgesic Effect on Neuropathic Pain of Retrogradely Transported botulinum Neurotoxin a Involves Schwann Cells and Astrocytes. PLoS ONE. 2012;7(10):e47977. 23110146 10.1371/journal.pone.0047977 PMC3480491

[257] Luvisetto S. Botulinum Neurotoxins in Central Nervous System: an Overview from Animal Models to Human Therapy. Toxins (Basel). 2021;13(11):751. 34822535 10.3390/toxins13110751 PMC8622321

[258] Popoff MR, Marvaud J. Structural and genomic features of clostridial neurotoxins. In: Alouf JE J Freer, editors. The Comprehensive Sourcebook of Bacterial Protein Toxins. London, UK: Academic Press; 1999. pp. 174–201.

[259] Doxey AC, Lynch MDJ, Müller KM, et al. Insights into the evolutionary origins of clostridial neurotoxins from analysis of the *Clostridium botulinum* strain a neurotoxin gene cluster. BMC Evol Biol. 2008;8(1):14. 19014598 10.1186/1471-2148-8-316 PMC2605760

[260] Popoff MR, Bouvet P. Genetic characteristics of toxigenic Clostridia and toxin gene evolution. Toxicon. 2013;75:63–89. 23707611 10.1016/j.toxicon.2013.05.003

[261] Nowakowska MB, Douillard FP, Lindström M. Looking for the X Factor in Bacterial Pathogenesis: association of orfX-p47 Gene Clusters with Toxin Genes in Clostridial and Non-Clostridial Bacterial Species. Toxins (Basel). 2019;12(1):19. 31906154 10.3390/toxins12010019 PMC7020563

[262] Dupuy B, Matamouros S. Regulation of toxin and bacteriocin synthesis in *Clostridium* species by a new subgroup of RNA polymerase σ-factors. Res Microbiol. 2006;157(3):201–205. 16439101 10.1016/j.resmic.2005.11.004

[263] Marvaud JC, Gibert M, Inoue K, et al. botR/A is a positive regulator of botulinum neurotoxin and associated non-toxin protein genes in *Clostridium botulinum* a. Mol Microbiol. 1998;29(4):1009–1018. 9767569 10.1046/j.1365-2958.1998.00985.x

[264] Raffestin S, Marvaud JC, Cerrato R, et al. Organization and regulation of the neurotoxin genes in *Clostridium botulinum* and *Clostridium tetani*. Anaerobe. 2004;10(2):93–100. 16701505 10.1016/j.anaerobe.2004.01.001

[265] Raffestin S, Dupuy B, Marvaud JC, et al. BotR/A and TetR are alternative RNA polymerase sigma factors controlling the expression of the neurotoxin and associated protein genes in *Clostridium botulinum* type a and *Clostridium tetani*. Mol Microbiol. 2004;55(1):235–249. 10.1111/j.1365-2958.2004.04377.x 15612931

[266] Dover N, Barash JR, Hill KK, et al. Novel Structural Elements within the Nonproteolytic *Clostridium botulinum* Type F Toxin Gene Cluster. Appl Environ Microbiol. 2011;77(5):1904. 21183631 10.1128/AEM.02422-10 PMC3067269

[267] Collins MD, East AK. Phylogeny and taxonomy of the food-borne pathogen *Clostridium botulinum* and its neurotoxins. J Appl Microbiol. 1998;84:5–17. 15244052 10.1046/j.1365-2672.1997.00313.x

[268] Smith TJ, Williamson CHD, Hill KK, et al. The Distinctive Evolution of orfX *Clostridium parabotulinum* Strains and Their Botulinum Neurotoxin Type a and F Gene Clusters is Influenced by Environmental Factors and Gene Interactions via Mobile Genetic Elements. Front Microbiol. 2021;12:566908. 33716993 10.3389/fmicb.2021.566908 PMC7952441

[269] Mansfield MJ, Adams JB, Doxey AC. Botulinum neurotoxin homologs in non-*Clostridium* species. FEBS Lett. 2015;589:342–348. 25541486 10.1016/j.febslet.2014.12.018

[270] Dover N, Barash JR, Burke JN, et al. Arrangement of the *Clostridium baratii* F7 Toxin Gene Cluster with Identification of a σ Factor That Recognizes the Botulinum Toxin Gene Cluster Promoters. PLoS ONE. 2014;9:e97983. 24853378 10.1371/journal.pone.0097983 PMC4031146

[271] Oguma K. The stability of toxigenicity in *Clostridium botulinum* types C and D. J Gen Microbiol. 1976;92(1):67–75. 1107486 10.1099/00221287-92-1-67

[272] Hariharan H, Mitchell WR. Observations on bacteriophages of *Clostridium botulinum* type C isolates from different sources and the role of certain phages in toxigenicity. Appl Environ Microbiol. 1976;32:145–158. 61735 10.1128/aem.32.1.145-158.1976 PMC170020

[273] Smith TJ, Hill KK, Foley BT, et al. Analysis of the Neurotoxin Complex Genes in Clostridium botulinum A1-A4 and B1 Strains: BoNT/A3, /Ba4 and /B1 Clusters Are Located within Plasmids. PLoS ONE. 2007;2(12):e1271. 10.1371/journal.pone.0001271 PMC2092393 18060065

[274] Marshall KM, Bradshaw M, Pellett S, et al. Plasmid Encoded Neurotoxin Genes in *Clostridium botulinum* Serotype a Subtypes. Biochem Biophys Res Commun. 2007;361:49. 17658467 10.1016/j.bbrc.2007.06.166 PMC2346372

[275] Franciosa G, Maugliani A, Scalfaro C, et al. Evidence that plasmid-borne botulinum neurotoxin type B genes are widespread among *Clostridium botulinum* serotype B strains. PLoS ONE. 2009;4(3):4. 10.1371/journal.pone.0004829 PMC2653641 19287483

[276] Zhang Z, Hintsa H, Chen Y, et al. Plasmid-Borne Type E Neurotoxin Gene Clusters in *Clostridium botulinum* Strains. Appl Environ Microbiol. 2013;79:3856. 23563942 10.1128/AEM.00080-13 PMC3675925

[277] Zhou Y, Sugiyama H, Nakano H, et al. The genes for the *Clostridium botulinum* type G toxin complex are on a plasmid. Infect Immun. 1995;63(5):2087. 7729925 10.1128/iai.63.5.2087-2091.1995 PMC173270

[278] Raphael BH, Bradshaw M, Kalb SR, et al. *Clostridium botulinum* Strains Producing BoNT/F4 or BoNT/F5. Appl Environ Microbiol. 2014;80:3250. 24632257 10.1128/AEM.00284-14 PMC4018930

[279] Dover N, Barash JR, Hill KK, et al. *Clostridium botulinum* Strain Af84 Contains Three Neurotoxin Gene Clusters: bont/A2, bont/F4 and bont/F5. PLoS ONE. 2013;8(4):e61205. 23637798 10.1371/journal.pone.0061205 PMC3625220

[280] Marshall KM, Bradshaw M, Johnson EA. Conjugative Botulinum Neurotoxin-Encoding Plasmids in *Clostridium botulinum*. PLoS ONE. 2010;5(6):e11087. 20552020 10.1371/journal.pone.0011087 PMC2884020

[281] Nawrocki EM, Bradshaw M, Johnson EA. Botulinum neurotoxin–encoding plasmids can be conjugatively transferred to diverse Clostridial strains. Sci Rep. 2018;8(1). 10.1038/s41598-018-21342-9 PMC5814558 29449580

[282] Smith TJ, Tian R, Imanian B, et al. Integration of Complete Plasmids Containing Bont Genes into Chromosomes of *Clostridium parabotulinum*, *Clostridium sporogenes*, and *Clostridium argentinense*. Toxins (Basel). 2021;13(7):473. 34357945 10.3390/toxins13070473 PMC8310154

[283] Dineen SS, Bradshaw M, Johnson EA. Neurotoxin gene clusters in *Clostridium botulinum* type a strains: sequence comparison and evolutionary implications. Curr Microbiol. 2003;46(5):345–352. 12732962 10.1007/s00284-002-3851-1

[284] Hill KK, Xie G, Foley BT, et al. Recombination and insertion events involving the botulinum neurotoxin complex genes in *Clostridium botulinum* types A, B, E and F and *Clostridium butyricum* type E strains. BMC Biol. 2009;7(1):66. 19804621 10.1186/1741-7007-7-66 PMC2764570

[285] Frankel G, Newton SMC, Schoolnik GK, et al. Intragenic recombination in a flagellin gene: characterization of the H1-j gene of *Salmonella typhi*. EMBO J. 1989;8(10):3149. 2583095 10.1002/j.1460-2075.1989.tb08468.x PMC401396

[286] Harrington CS, Thomson-Carter FM, Carter PE. Evidence for recombination in the flagellin locus of *Campylobacter jejuni*: implications for the flagellin gene typing scheme. J Clin Microbiol. 1997;35(9):2386–2392. 9276421 10.1128/jcm.35.9.2386-2392.1997 PMC229973

[287] Carter AT, Paul CJ, Mason DR, et al. Independent evolution of neurotoxin and flagellar genetic loci in proteolytic *Clostridium botulinum*. BMC Genomics. 2009;10(1):115. 19298644 10.1186/1471-2164-10-115 PMC2674064

[288] Bradshaw M, Dineen SS, Maks ND, et al. Regulation of neurotoxin complex expression in *Clostridium botulinum* strains 62A, Hall A-hyper, and NCTC 2916. Anaerobe. 2004;10(6):321–333. 16701534 10.1016/j.anaerobe.2004.07.001

[289] Bonventre PF, Kempe LL. Physiology of toxin production by *Clostridium botulinum* types a and B. I. Growth, autolysis, and toxin production. J Bacteriol. 1960;79:18–23. 13802633 10.1128/jb.79.1.18-23.1960 PMC278628

[290] Couesnon A, Raffestin S, Popoff MR. Expression of botulinum neurotoxins a and E, and associated non-toxin genes, during the transition phase and stability at high temperature: analysis by quantitative reverse transcription-PCR. Microbiology. 2006;152(3):759–770. 16514155 10.1099/mic.0.28561-0

[291] Ihekwaba AEC, Mura I, Malakar PK, et al. New elements to consider when modeling the hazards associated with botulinum neurotoxin in food. J Bacteriol. 2016;198(2):204–211. 26350137 10.1128/JB.00630-15 PMC4751798

[292] Connan C, Denève C, Mazuet C, et al. Regulation of toxin synthesis in C*lostridium botulinum* and *Clostridium tetani*. Toxicon. 2013;75:90–100. 23769754 10.1016/j.toxicon.2013.06.001

[293] Cooksley CM, Davis IJ, Winzer K, et al. Regulation of neurotoxin production and sporulation by a putative agrBD signaling system in proteolytic *Clostridium botulinum*. Appl Environ Microbiol. 2010;76(13):4448–4460. 20453132 10.1128/AEM.03038-09 PMC2897414

[294] Zhang Z, Korkeala H, Dahlsten E, et al. Two-Component Signal Transduction System CBO0787/CBO0786 Represses Transcription from Botulinum Neurotoxin Promoters in *Clostridium botulinum* ATCC 3502. PLOS Pathogens. 2013;9(3):e1003252. 23555260 10.1371/journal.ppat.1003252 PMC3610760

[295] Connan C, Popoff MR. Two-component systems and toxinogenesis regulation in *Clostridium botulinum*. Res Microbiol. 2015;166(4):332–343. 25592073 10.1016/j.resmic.2014.12.012

[296] Bonventre PF, Kempe LL. Physiology of toxin production by *Clostridium botulinum* types a and B. III. Effect of pH and temperature during incubation on growth, autolysis. and toxin production. Appl Microbiol. 1959;7(6):374–377. 13802635 10.1128/am.7.6.374-377.1959 PMC1057564

[297] Leyer GJ, Johnson EA. Repression of toxin production by tryptophan in *Clostridium botulinum* type E. Arch Microbiol. 1990;154(5):443–447. 2256780 10.1007/BF00245225

[298] Fredrick CM, Lin G, Johnson EA. Regulation of botulinum neurotoxin synthesis and toxin complex formation by arginine and glucose in *Clostridium botulinum* ATCC 3502. Appl Environ Microbiol. 2017;83(13). 10.1128/AEM.00642-17 PMC5479000 28455330

[299] Bowers LE, Williams OB. Effect of arginine on growth and lysis of *Clostridium botulinum*. J Bacteriol. 1963;85:1175. 14044015 10.1128/jb.85.5.1175-1176.1963 PMC278304

[300] Inzalaco HN, Tepp WH, Fredrick C, et al. Posttranslational Regulation of Botulinum Neurotoxin Production in *Clostridium botulinum* Hall A- hyper. mSphere. 2021;6(4):6. 10.1128/mSphere.00328-21 PMC8386421 34346710

[301] Zhang Z, Dahlsten E, Korkeala H, et al. Positive regulation of botulinum neurotoxin gene expression by CodY in *Clostridium botulinum* ATCC 3502. Appl Environ Microbiol. 2014;80:7651–7658. 25281376 10.1128/AEM.02838-14 PMC4249235

[302] Ihekwaba AEC, Mura I, Walshaw J, et al. An Integrative Approach to Computational Modelling of the Gene Regulatory Network Controlling *Clostridium botulinum* Type A1 Toxin Production. PLoS Comput Biol. 2016;12(11):e1005205. 27855161 10.1371/journal.pcbi.1005205 PMC5113860

[303] Piggot PJ, Hilbert DW. Sporulation of *Bacillus subtilis*. Curr Opin Microbiol. 2004;7:579–586. 15556029 10.1016/j.mib.2004.10.001

[304] Paredes CJ, Alsaker KV, Papoutsakis ET. A comparative genomic view of clostridial sporulation and physiology. Nature Rev Microbiol. 2005;3(12):969–978. 16261177 10.1038/nrmicro1288

[305] Tan IS, Ramamurthi KS. Spore formation in *Bacillus subtilis*. Environ Microbiol Rep. 2014;6:212–225. 24983526 10.1111/1758-2229.12130 PMC4078662

[306] Wörner K, Szurmant H, Chiang C, et al. Phosphorylation and functional analysis of the sporulation initiation factor Spo0A from *Clostridium botulinum*. Mol Microbiol. 2006;59:1000–1012. 16420367 10.1111/j.1365-2958.2005.04988.x

[307] Davidson P, Eutsey R, Redler B, et al. Flexibility and constraint: evolutionary remodeling of the sporulation initiation pathway in Firmicutes. PLoS Genet. 2018;14(9):e1007470. 30212463 10.1371/journal.pgen.1007470 PMC6136694

[308] Hoch JA. Regulation of the phosphorelay and the initiation of sporulation in *Bacillus subtilis*. Annu Rev Microbiol. 1993;47(1):441–465. 8257105 10.1146/annurev.mi.47.100193.002301

[309] Kirk DG, Zhang Z, Korkeala H, et al. Alternative sigma factors SigF, SigE, and SigG are essential for sporulation in *Clostridium botulinum* ATCC 3502. Appl Environ Microbiol. 2014;80:5141–5150. 24928875 10.1128/AEM.01015-14 PMC4135750

[310] Kirk DG, Palonen E, Korkeala H, et al. Evaluation of normalization reference genes for RT-Qpcr analysis of spo0A and four sporulation sigma factor genes in *Clostridium botulinum* Group I strain ATCC 3502. Anaerobe. 2014;26:14–19. 24389585 10.1016/j.anaerobe.2013.12.003

[311] Mascher G, Mertaoja A, Korkeala H, et al. Neurotoxin synthesis is positively regulated by the sporulation transcription factor Spo0A in *Clostridium botulinum* type E. Environ Microbiol. 2017;19(10):4287–4300. 28809452 10.1111/1462-2920.13892

[312] Imae Y, Strominger JL. Cortex content of asporogenous mutants of *Bacillus subtilis*. J Bacteriol. 1976;126(2):914–918. 1262319 10.1128/jb.126.2.914-918.1976 PMC233229

[313] Setlow P. Spores of *Bacillus subtilis*: their resistance to and killing by radiation, heat and chemicals. J Appl Microbiol. 2006;101(3):514–525. 16907802 10.1111/j.1365-2672.2005.02736.x

[314] Portinha IM, Douillard FP, Korkeala H, et al. Sporulation Strategies and Potential Role of the Exosporium in Survival and Persistence of *Clostridium botulinum*. Int J Mol Sci. 2022;23(2):23. 10.3390/ijms23020754 PMC8775613 35054941

[315] Setlow P. Germination of spores of *Bacillus* species: what we know and do not know. J Bacteriol. 2014;196:1297–1305. 24488313 10.1128/JB.01455-13 PMC3993344

[316] Moir A. How do spores germinate? J Appl Microbiol. 2006;101:526–530. 16907803 10.1111/j.1365-2672.2006.02885.x

[317] Brunt J, van Vliet AHM, van den Bos F, et al. Diversity of the germination apparatus in *Clostridium botulinum* groups I, II, III, and IV. Front Microbiol. 2016;7:1702. DOI:10.3389/fmicb.2016.01702. 27840626 PMC5083711

[318] Clauwers C, Lood C, Van Noort V, et al. Canonical germinant receptor is dispensable for spore germination in *Clostridium botulinum* group II strain NCTC 11219. Sci Rep. 2017;7:1–10. 29133849 10.1038/s41598-017-15839-y PMC5684421

[319] Ross C, Abel-Santos E. The Ger Receptor Family from Sporulating Bacteria. Curr Issues Mol Biol. 2010;12:147. 20472940 PMC3081667

[320] Brunt J, Carter AT, Pye HV, et al. The orphan germinant receptor protein GerXAO (but not GerX3b) is essential for L-alanine induced germination in *Clostridium botulinum* Group II. Sci Rep. 2018;8(1):1–10. 29728678 10.1038/s41598-018-25411-x PMC5935672

[321] Cañadas IC, Groothuis D, Zygouropoulou M, et al. RiboCas: a Universal CRISPR-Based Editing Tool for *Clostridium*. ACS Synth Biol. 2019;8(6):1379–1390. 31181894 10.1021/acssynbio.9b00075

[322] Mertaoja A, Nowakowska MB, Mascher G, et al. CRISPR-Cas9-Based Toolkit for *Clostridium botulinum* Group II Spore and Sporulation Research. Front Microbiol. 2021;12:32. 10.3389/fmicb.2021.617269 PMC7873358 33584620

[323] Heap JT, Pennington OJ, Cartman ST, et al. The ClosTron: a universal gene knock-out system for the genus *Clostridium*. J Microbiol Methods. 2007;70:452–464. 17658189 10.1016/j.mimet.2007.05.021

[324] Heap JT, Ehsaan M, Cooksley CM, et al. Integration of DNA into bacterial chromosomes from plasmids without a counter-selection marker. Nucleic Acids Res. 2012;40(8):e59. 22259038 10.1093/nar/gkr1321 PMC3333862

[325] Nowakowska MB, Selby K, Przykopanski A, et al. Construction and validation of safe *Clostridium botulinum* Group II surrogate strain producing inactive botulinum neurotoxin type E toxoid. Sci Rep. 2022;12(1):1790. 35110559 10.1038/s41598-022-05008-1 PMC8810926

[326] Chen S, Barbieri JT. Engineering botulinum neurotoxin to extend therapeutic intervention. Proc Natl Acad Sci, USA. 2009;106:9180–9184. 19487672 10.1073/pnas.0903111106 PMC2695098

[327] Vazquez-Cintron EJ, Vakulenko M, Band PA, et al. Atoxic derivative of botulinum neurotoxin a as a prototype molecular vehicle for targeted delivery to the neuronal cytoplasm. PLoS ONE. 2014;9(1):e85517. 24465585 10.1371/journal.pone.0085517 PMC3899041

[328] Vazquez-Cintron E, Tenezaca L, Angeles C, et al. Pre-clinical study of a novel recombinant botulinum neurotoxin derivative engineered for improved safety. Sci Rep. 2016;6:6. 27484492 10.1038/srep30429 PMC4971498

[329] Tao L, Peng L, Berntsson RP-A, et al. Engineered botulinum neurotoxin B with improved efficacy for targeting human receptors. Nat Commun. 2017; 8(1). 10.1038/s41467-017-00064-y PMC5495808 28674381

[330] Guo J, Pan X, Zhao Y, et al. Engineering clostridia neurotoxins with elevated catalytic activity. Toxicon. 2013;74:158–166. 23994593 10.1016/j.toxicon.2013.08.055

